# The landscape of cancer-rewired GPCR signaling axes

**DOI:** 10.1016/j.xgen.2024.100557

**Published:** 2024-05-08

**Authors:** Chakit Arora, Marin Matic, Luisa Bisceglia, Pierluigi Di Chiaro, Natalia De Oliveira Rosa, Francesco Carli, Lauren Clubb, Lorenzo Amir Nemati Fard, Giorgos Kargas, Giuseppe R. Diaferia, Ranka Vukotic, Luana Licata, Guanming Wu, Gioacchino Natoli, J. Silvio Gutkind, Francesco Raimondi

**Affiliations:** 1 Laboratorio di Biologia Bio@SNS, Scuola Normale Superiore, Piazza dei Cavalieri 7, 56126 Pisa, Italy; 2 Department of Experimental Oncology, IEO, European Institute of Oncology IRCCS, Milano, Italy; 3 Department of Pharmacology and Moores Cancer Center, University of California, San Diego, La Jolla, CA 92093, USA; 4 Azienda Ospedaliero-Universitaria Pisana, Via Roma, 67, 56126 Pisa, Italy; 5 Department of Biology, University of Rome Tor Vergata, 00133 Rome, Italy; 6 Division of Bioinformatics and Computational Biology, Department of Medical Informatics and Clinical Epidemiology, Oregon Health & Science University, Portland, OR, USA

**Keywords:** GPCR, cancer, transcriptomics, cell-cell communication, personalized medicine, cancer cell lines, survival analysis, drug repurposing, signaling network

## Abstract

We explored the dysregulation of G-protein-coupled receptor (GPCR) ligand systems in cancer transcriptomics datasets to uncover new therapeutics opportunities in oncology. We derived an interaction network of receptors with ligands and their biosynthetic enzymes. Multiple GPCRs are differentially regulated together with their upstream partners across cancer subtypes and are associated to specific transcriptional programs and to patient survival patterns. The expression of both receptor-ligand (or enzymes) partners improved patient stratification, suggesting a synergistic role for the activation of GPCR networks in modulating cancer phenotypes. Remarkably, we identified many such axes across several cancer molecular subtypes, including many involving receptor-biosynthetic enzymes for neurotransmitters. We found that GPCRs from these actionable axes, including, e.g., muscarinic, adenosine, 5-hydroxytryptamine, and chemokine receptors, are the targets of multiple drugs displaying anti-growth effects in large-scale, cancer cell drug screens, which we further validated. We have made the results generated in this study freely available through a webapp (gpcrcanceraxes.bioinfolab.sns.it).

## Highlights

• Systematic analysis of GPCR extracellular network in cancer transcriptomics datasets
• Multiple GPCR-ligand and biosynthetic enzymes axes are deregulated in cancer
• GPCR signaling axes expression correlates with patient survival
• Drug targeting of GPCR axes inhibits the growth of cancer cell lines

## Graphical abstract

**Figure:**
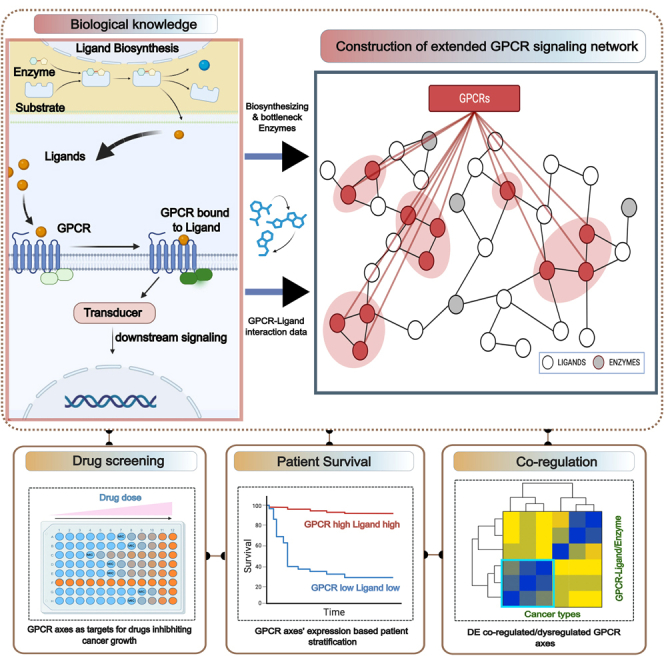

## Teaser

Arora et al. provide a comprehensive computational biology analysis of GPCR extracellular networks in cancer transcriptomics datasets, which reveals signaling axes associated to patient survival. Drug targeting of GPCR axes is associated with growth inhibition of cancer cell lines, thus offering new drug repurposing opportunities in oncology.

## Introduction

G-protein-coupled receptors (GPCRs) are the largest family of transmembrane proteins and transduce a wide range of physical and chemical stimuli into the cell by coupling to intracellular heterotrimeric G proteins, thereby controlling multiple downstream signaling pathways and transcriptional programs.^1^^,^^2^^,^^3^ The GPCR repertoire expanded and diversified to meet the increasing need for inter-cellular communication in evolving multicellular organisms.^4^^,^^5^ Systematic efforts are being undertaken to illuminate the complex circuitry of GPCR signaling both at the intracellular^5^^,^^6^^,^^7^^,^^8^^,^^9^^,^^10^^,^^11^ as well as the extracellular ligand level.^12^

Cancer genomics provides the possibility to systematically shed light on cancer somatic alterations affecting GPCRs and their interacting partners.^13^^,^^14^^,^^15^^,^^16^^,^^17^ Indeed, a landscape of GPCR-centric autocrine, paracrine, juxtacrine, or endocrine signaling networks is emerging as a critical component for the interaction of cancer cells with their tumor micro-environment (TME), ultimately leading to cancer progression and therapy resistance.^15^^,^^16^ For instance, chemokines, such as *CXCL1*, *CXCL2*, and *CXCL8*, bind and activate their receptors, primarily *CXCR1* and *CXCR2* on immune suppressive myeloid derived cells, thereby disabling anti-tumoral immune surveillance mechanisms.^15^ Several GPCRs and their associated components mediating inflammation have also been connected to immune evasive TME, such as prostaglandins (in particular *PGE2*), their biosynthesizing enzymes (*PTGS1* and *PTGS2*), and cognate prostanoid receptors (e.g., *PTGER2* and *PTGER4*).^15^ Ectonucleotidases (such as *NT5E* or *ENTPD1*) and adenosine receptors are also well known to induce an immunosuppressive TME.^15^ Recent studies have also shown the emergent role of innervation in promoting tumor growth (reviewed in Hanahan and Monje^18^). Notably, it has emerged that there is a crosstalk between the neurotrophin pathway, particularly the nerve growth factor (*NGF)-NTRK1* axes, and GPCR signaling mediated by either (nor)adrenaline-*ADRB2* in pancreatic cancer,^19^ or acetylcholine-*CHRM1* and *CHRM3* in gastric cancer.^20^ The neuron-cancer cell communication can also be established via additional routes, such as the one of 5-hydroxytryptamine, which is produced by enteric serotonergic neurons, and activates cognate receptors expressed on colorectal cancer stem cells, which undergo self-renewal and tumorigenesis.^21^ However, defining these immune-evasive and tumor-promoting networks has been elusive. This is also due to the interaction complexity arising from the two key molecular recognition steps governing GPCRs’ function; i.e., the binding of extracellular ligands and the engagement and activation of intracellular transducers, both of which are frequently dysregulated in cancer.

In this regard, single-cell and spatial sequencing technologies are enabling the study of mechanisms of cell-cell communications at an unprecedented resolution.^22^ In the most comprehensive resource to date used to infer ligand-receptor pairs mediating cell-cell communication,^23^ GPCRs-ligand interactions are surprisingly depleted, representing roughly less than 5% of the total curated interactions, despite being the most abundant class of transmembrane proteins involved in signal transmission. This is probably because GPCRs typically interact with small organic molecules, such as neurotransmitters and various metabolites, whose expression is not detectable by transcriptomics techniques, thus preventing the identification of such interactions by canonical cell-cell communication algorithms.

In the present study, we considered an extended network of signaling axes formed by GPCRs with interacting ligands, as well as biosynthetic enzyme pathways, to systematically explore their aberrant regulation in cancer transcriptomics datasets and investigate their value for patient stratification for personalized medicine treatments.

## Results

### GPCRs extracellular signaling network and associated onco-programs

**Figure 1:**
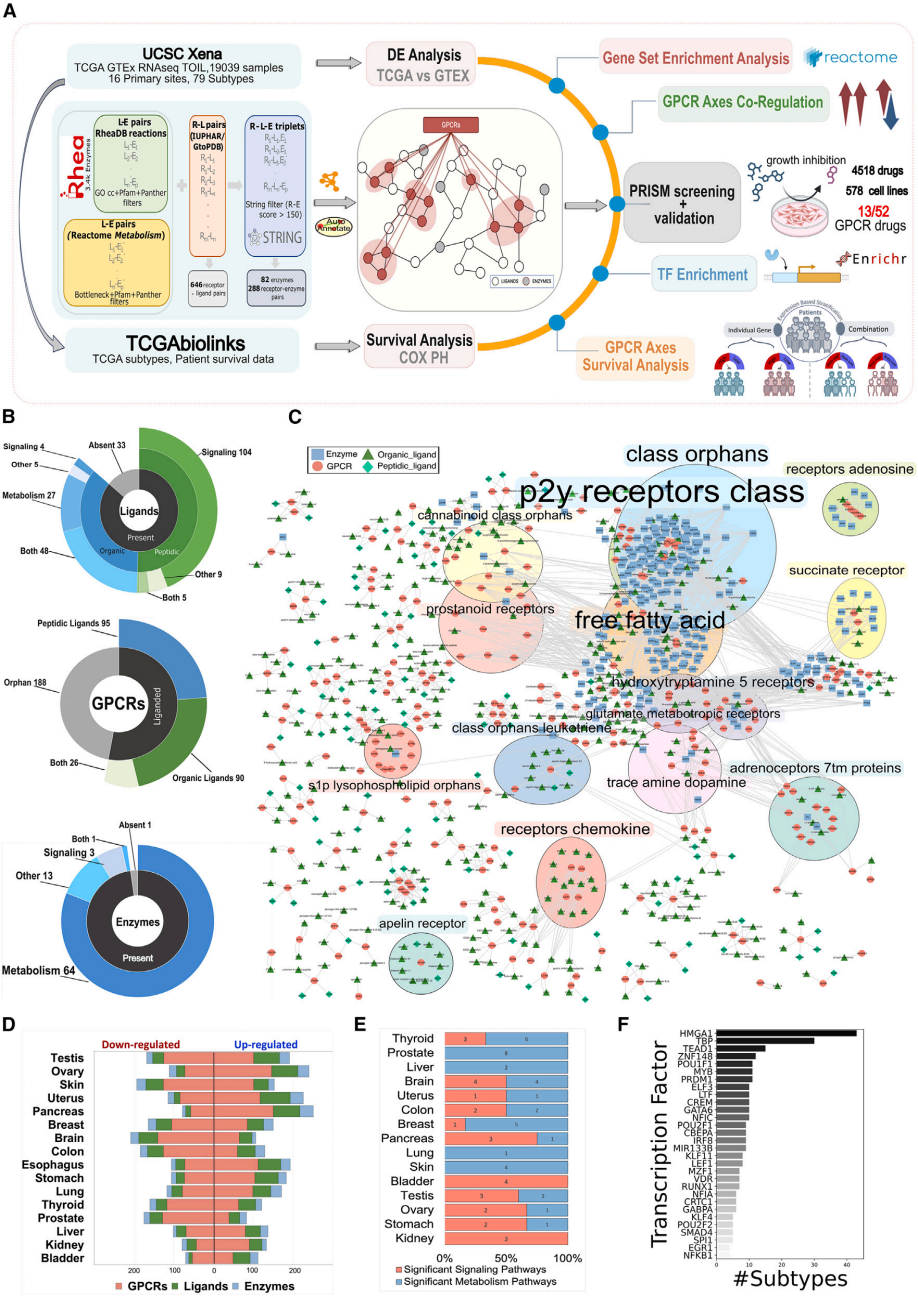
GPCR extracellular signaling network and overview of the datasets considered (A) Workflow of the study involving the procedure to generate the interacting enzyme-receptor pairs (left center) to generate the GPCR-ligand-enzyme extended network (center), the dataset employed (left top and bottom) to perform the DE and survival analysis (center top and bottom) and subsequently the downstream analysis steps (right). (B) Sunburst charts providing an overview of the number of liganded and orphan G-protein-coupled receptors (GPCRs) considered; classification of the liganded GPCRs based on their ligand type, i.e., either peptide, organic, or a combination of both; number of enzymes that are either currently included or absent within the Reactome pathways. The included ligands are additionally subcategorized based on the frequency of their types and the frequency of the Reactome pathway domains with which they are linked. For enzymes, only the second layer of this information is shown. (C) The network of GPCRs and cognate ligands and biosynthetic enzymes. (D) Funnel plot exhibiting the frequency of differentially expressed (TCGA vs. GTEx, BH-corrected adjusted *p* <0.01) GPCRs, ligands, and enzymes corresponding to each whole tissue. (E) Stacked bar plot displaying the number of significantly enriched ligand-associated pathways within Reactome sub-domains: signaling (R-HSA-372790: signaling by GPCR) and metabolism (R-HSA-1430728:metabolism). Each bar represents the relative proportions of different categories as percentages of the whole, with respect to each tissue. An enriched pathway refers to a pathway enriched in TCGA with a GSEA enrichment score >0 and BH-corrected adjusted *p* <0.01. (F) The bar plot illustrates the top 30 enriched transcription factors, with the y axis representing the transcription factors and the x axis indicating the count of subtypes in which they are enriched.

We first generated a comprehensive catalog of upstream and downstream molecular interactions involving GPCRs (Figures 1A–1C; see STAR Methods). Briefly, we retrieved known GPCR ligands from IUPHAR (International Union of Basic and Clinical Pharmacology)/Guide to Pharmacology database (GtoPDB), which we mapped to individual ligand-synthesizing enzymes and to known biosynthetic pathways, as well as to pathways mediating signal transduction (STAR Methods; Tables S1, and S2). This yielded a total of 236 ligands, 309 biosynthetic pathways, and 82 enzyme-associated instances (Figures 1A–1C). We found that 86% of ligands and 99% of enzymes were already annotated within biological pathway knowledgebases (i.e., Reactome; Figure 1B). A total of 211 (53%) non-olfactory receptors have known ligands in GtoPdb, 95 of which exclusively bind organic ligands, 90 peptides, and 26 both ligand types (Figure 1B). While the vast majority of peptide ligands are annotated in signaling pathways, the organic ligands are instead extensively annotated as part of metabolic processes (Figure 1B, and Table S1).

We then computed genome-wide differential expression (DE) by contrasting cancer samples from The Cancer Genome Atlas (TCGA) project to those from corresponding healthy tissues from the Genotype-Tissue Expression (GTEx) project to derive expression signatures of genes and pathways mediating GPCR signaling. We considered a total of 15,000 samples (10,500 from TCGA and 4,500 from GTEx), encompassing 16 tissues, as well as 79 cancer molecular subtypes (see STAR Methods; Table S3).

Remarkably, we found a widespread, cancer-specific regulation of GPCR-related genes with respect to the corresponding healthy tissues. We found, on average, 91 upregulated and 92 downregulated GPCRs (false discovery rate [FDR] <0.01, |log2FC| > 1) per cancer. When considered together with their cognate ligands and biosynthetic enzymes, we obtained an average of 161 upregulated and 133 downregulated instances (Figure 1D). The amount of significantly differentially expressed genes varies across tissues, with testis, skin, and ovary being the most affected, and bladder the least (Figure 1D). Certain cancer tissues are characterized by a prevalence of either significantly over-expressed (e.g., pancreas) or downregulated (e.g., brain) GPCRs (Figure S1A). We found no significant trend when grouping differentially expressed receptors based on their G-protein coupling specificity (Figure S1A). On the other hand, DE of heterotrimeric G-protein α subunits showed that *GNAS* was the single most widely upregulated subunit, being significantly over-expressed in 94% of cancer tissues (Figures S1A, and S1C), confirming previous analyses.^15^^,^^17^

At the process level, we found a widespread DE regulation (FDR < 0.01) of GPCR pathways across cancers (Figure 1E). In particular, 40% of cancer tissues are either more enriched in biosynthetic or signal transduction processes, while 20% of cancer tissues are equally affected in both (Figure 1E). Several pathways are found to be extensively affected (e.g., upregulation of chemokine receptors), while others are exclusively regulated in certain tumor types (Figure S1D). Overall, these data show that every cancer tissue is characterized by highly specific GPCR pathway signatures (Figure S1D). Analysis of the over-representation of genes regulating transcriptional activity for differentially expressed GPCRs further emphasized that each cancer subtype displays specific transcriptional programs significantly associated (FDR < 0.05) with differentially expressed GPCRs (Figures 1F, and S2; see STAR Methods). For example, we found that certain genes, such as *HMGA1* or *TEAD1*, are recurrently associated with GPCRs' expression across multiple tumor subtypes (Figure 1F). Gastro-intestinal tract tumor types, such as the stomach subtypes “chromosomal instability” (Stomach_GI.CIN) or “genomically stable” (Stomach_GI.GS), display the highest number of transcriptional regulators associated with differentially expressed GPCRs (Figure S2).

### The landscape of peptide ligand-GPCRs dysregulation in cancer

We focused our analysis on the extracellular components of the GPCR signaling machinery (e.g., ligands and related metabolizing enzymes) to understand their contribution to the overall dysregulation of GPCR signaling in cancer.

We found, on average, 66 significantly differentially expressed peptide ligand precursors per cancer in 16 cancer tissues, and, on average, 60 instances per cancer in 79 molecular subtypes.

**Figure 2:**
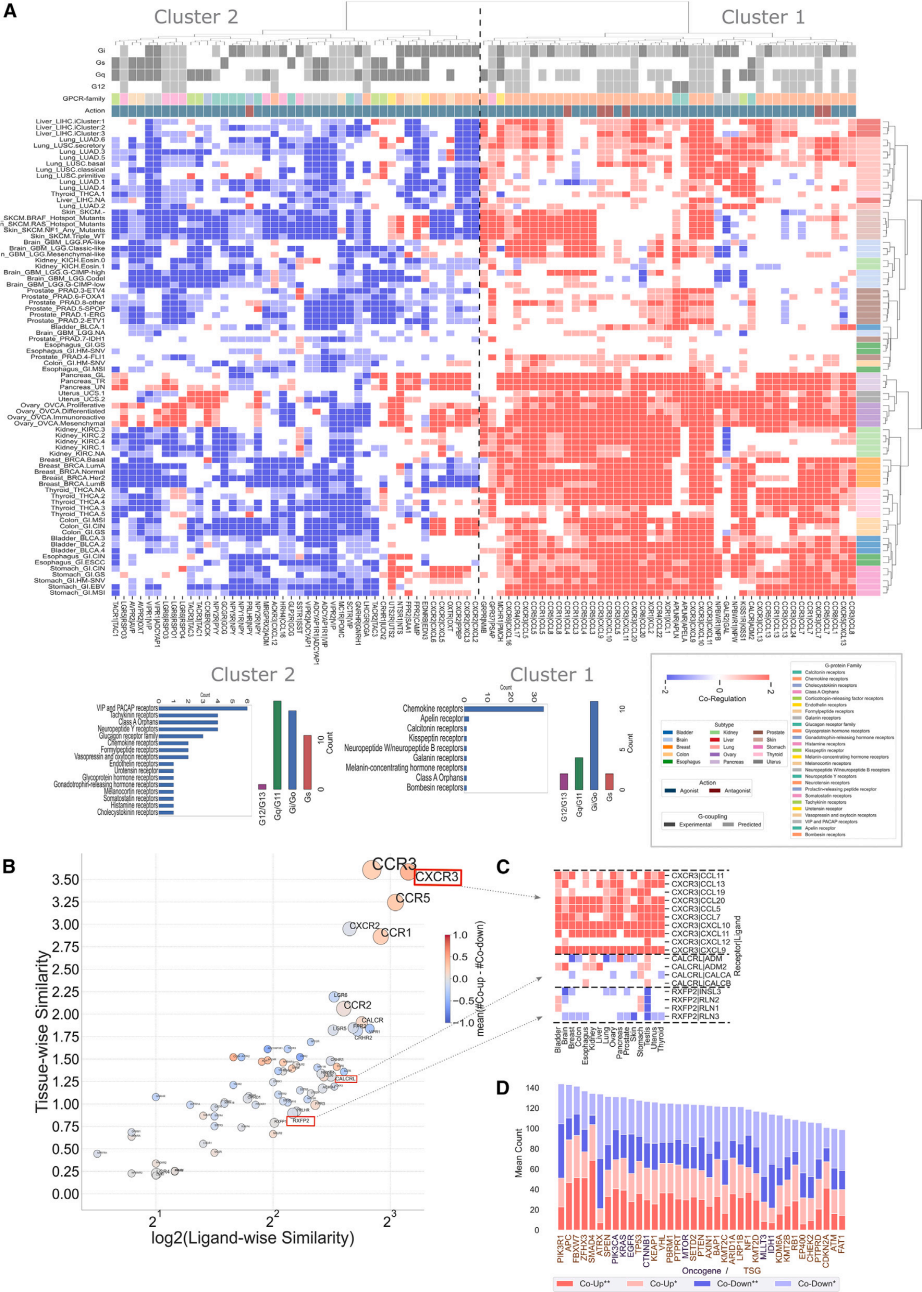
Peptide ligand-receptor co-regulation (A) Heatmap (center panel) displaying the co-differential regulation for receptor-ligand pairs across different TCGA subtypes (color coded at the top row). Darker red represents both receptor and ligand significantly co-upregulated in TCGA (i.e., log2-fold change, LFC > 1 and adjusted *p* <0.01) and darker blue represents both receptor and ligand significantly co-downregulated in TCGA (i.e., LFC < 1 and adjusted *p* <0.01). Paler colors represent either the receptor or ligand as significantly differentially expressed (i.e., |LFC| > 1 for both but adjusted *p* < 0.01 for only one of these). White cells indicate anti-regulation or no fold change at all in at least one of them. Only those pairs that are affected in at least 25% of TCGA subtypes are displayed. A dashed line separating the two clusters, created using Hierarchical clustering, is shown in the middle. Hierarchical clustering was performed using the Ward method to identify two homogeneous clusters by minimizing within-cluster variance based on the sum of squared differences of feature values; i.e., scores∈[−2, + 2] assigned to each pair. Heatmap (left panel) uses color codes to display the ligand’s mechanism of action in “Action,” G-protein coupling associated with the GPCRs, and GPCR family. Heatmap (right panel) displays the information contained in the left panel as concise bar plots for each of the two clusters. For each cluster, bar plots indicate G-protein coupling preferences as well as GPCR families represented as count of the number of receptors. (B) The scatterplot displays a GPCR-centric view of the heatmap in (A) based on tissue-wise or ligand-wise similarity. Here, ligand-wise similarity for each GPCR is the average of the pairwise Euclidean distances between co-differential expression (co-DE) profiles across its ligands (i.e., vertically), while tissue-wise similarity is a similar metric across tissues (i.e., horizontally). The size of the markers is proportional to the number of ligands, and the colors correspond to the average difference in the number of co-up and co-down profiles. GPCRs located in the top-right corner are more diverse in terms of their ligand interactions. (C) Diverse co-DE profiles are observed with respect to different ligands and tissues corresponding to the same GPCR. For example, CXCR3 and its corresponding ligands exhibit an almost exclusively co-upregulated profile irrespective of tissue type, while CALCRL and its ligands display varied co-regulation profiles, implying a varying interaction with its ligands in different tissue (or cancer) types. (D) The bar plot visually presents the ranking of oncogenes based on the average count of co-regulated GPCR-ligand instances across subtypes. The x axis denotes individual oncogenes/tumor suppressor genes (TSGs), color-coded separately for clarity, while the y axis represents the corresponding average count.

We next assessed the degree of correlation of expression changes between receptors and interacting ligands and found a widespread co-regulation of receptor-ligand precursor pairs in multiple cancer tissues and subtypes (Figures 2A, and S3; see STAR Methods). By focusing on the receptor-ligand pairs more recurrently co-regulated, we identified two clusters, showing respectively a prevalence of concordantly upregulated (i.e., cluster 1) and concordantly downregulated (i.e., cluster 2) axes across subtypes (Figure 2A). The majority of the regulated ligands are agonists, except for *CCL4*; *CCL7*; and *CXCL8*, *10*, and *11*, which might act as either agonists or antagonists depending on the bound receptor (Figure 2A). Most axes in cluster 1 involve chemokine receptors (e.g., *CCR1*, *CCR3*, *CCR4*, *CCR5*, *CCR6*, *CCR8*, *CXCR3*, *CXCR6*, *XCR1*) as well as other receptors such as *APLNR*, *CALCR*, *GRPR*, *MCHR1*, *GPR37*, *CCKBR*, *NPBWR1*, *KISS1R*, and their cognate ligands. The main transduction mechanism in this group is through G_i/o_ proteins, as well as to a lesser extent via G_q/11_ and G_12/13_ (Figure 2A).

Cluster 2 contains instead receptor-ligand axes that are more frequently concordantly downregulated in cancer subtypes, although several of these might still be concordantly upregulated in specific subtypes (e.g., *CXCR2*, *EDNRB*, *FFPR2*, or *LGR6*, *GALR2*, and *UTS2R*; Figure 2A). The axes in cluster 2 involve receptors that are members of various families from class A and B. Receptors in this group have a more heterogeneous/promiscuous coupling profile to G_q/11_, G_i/o_, and G_s_, but very little to G_12/13_ (Figure 2A).

In general, every cancer subtype is characterized by a highly specific receptor-ligand co-expression signature. However, certain tissues display similar subtype co-expression patterns (e.g., liver, breast, ovary, thyroid, stomach, lung, skin, and pancreas). Others show higher heterogeneity (e.g., kidney, colon, bladder, esophagus, prostate, brain) (Figure 2A). We also assessed the similarities of GPCR DE profiles across tissues and ligands (see STAR Methods), which revealed that co-regulation patterns of a given receptor can vary on a tissue and ligand basis (Figures 2B, and S3). Certain receptors display consistent co-regulation patterns across cancer tissues and subtypes even if bound to multiple ligands (e.g., *CXCR3*, *CCR3*, *CCR5*; Figures 2B and 2C). Others show more variable co-regulation patterns that vary in a tissue (or subtype) and ligand specific fashion (e.g., *CALCRL* or *RXFP2*; Figures 2B and 2C). Strikingly, we found that 73 out of 79 cancer subtypes (92.4%) are characterized by a prevalence of concordant (i.e., having the same log2-fold change (LFC) directionality) vs. discordant (i.e., having opposite LFC directionality) regulation of GPCR axes, suggesting tight correlated expression of the components of the same receptor-ligand axis (Figure S4).

We associated the most frequently mutated cancer genes in each TCGA subtype with the presence of up- or downregulated GPCR axes (Figure S5; see STAR Methods). Based on cancer gene mutation clustering, we could find a group of subtypes (mainly prostate and brain tumors) that are characterized by overall co-downregulation of GPCR axes and concomitant mutations of several tumor suppressor genes (TSGs). G-protein couplings of concordantly downregulated axes are transversely decreased. In the concordantly upregulated axes, we found instead much variability, with G_12/13_ prevailing, relative to co-downregulated axes, as well as G_s_ completely lacking in certain tumors (Figure S5A–S5E). Cancer subtypes with G_12/13_ prevalence in the concordantly upregulated axes are characterized by mutations of the *KRAS*, *PIK3CA*, and *MLLT3* oncogenes (Figure S5E, and Table S4). By ranking cancer genes based on the average count of co-regulated GPCR-ligand instances across subtypes, we found that the majority of top associated cancer genes are TSGs (seven out of the top 10). Interestingly, *APC*, *FBXW7*, *ZFHX3*, and *SMAD4* are all associated with a prevalence of concordantly upregulated signaling axes, while *PIK3R1* or *ATRX* are overall associated with concordantly downregulated GPCRs (Figure 2D).

### Expression of biosynthetic pathway enzymes inform on the levels of organic endogenous ligands

We reasoned that the expression levels of the enzymes synthesizing organic GPCR ligands could be informative of their levels and thus be used as a proxy to model ligand abundance in transcriptomics datasets. To this end, we assessed enzyme and biosynthetic pathway activities from differential gene expression signatures (see STAR Methods).

**Figure 3:**
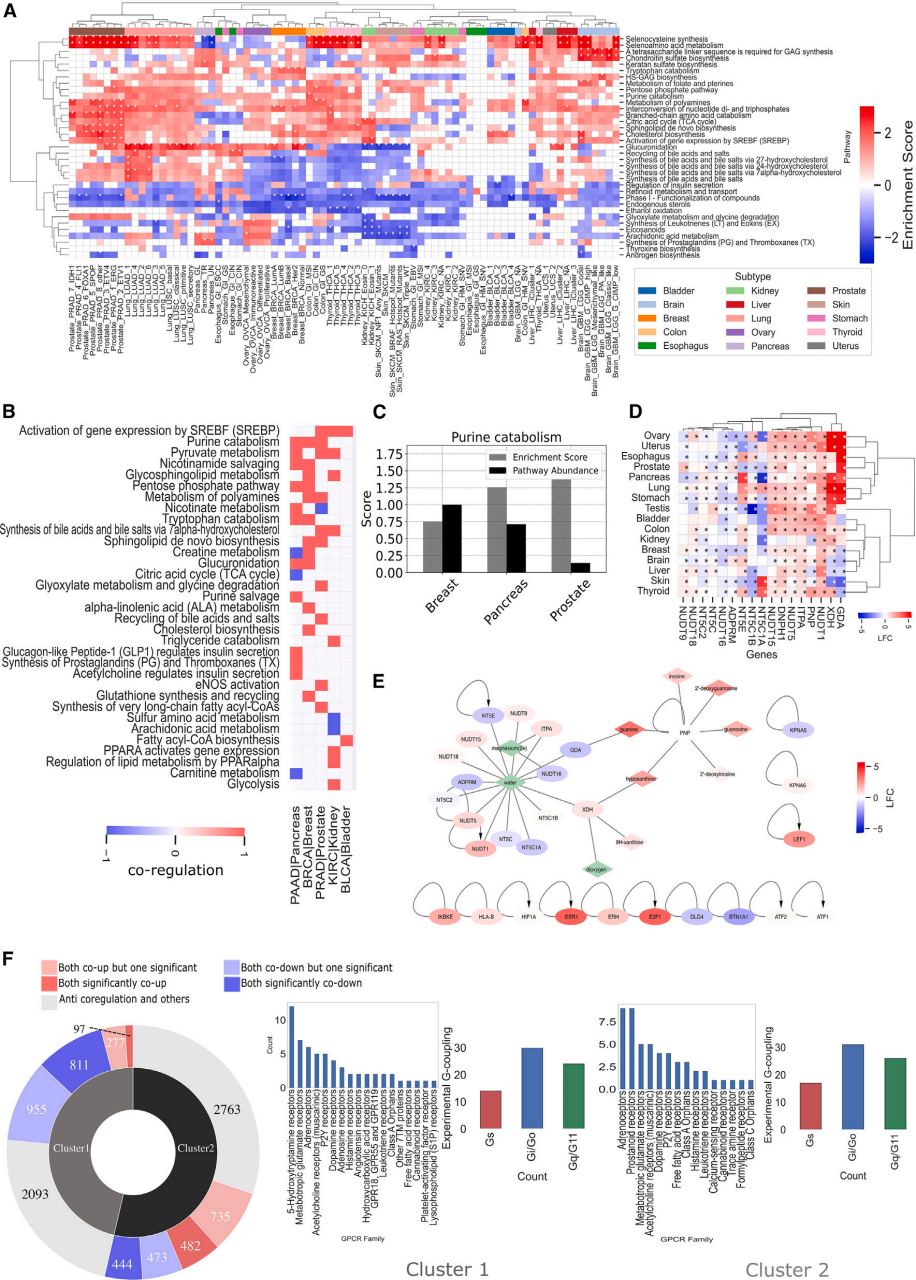
Biosynthetic pathway enrichment (A) Heatmap of GSEA pathway enrichment analysis for differentially expressed genes in cancer (TCGA) over normal (GTEx) samples, considering Reactome pathways related to signaling and metabolism. Red cells indicate pathways enriched in TCGA, blue cells indicate pathways enriched in GTEx, and white cells indicate no enrichment. Cells marked with an asterisk (∗) correspond to a statistically significant enrichment (adjusted *p* <0.01). (B) Concordant pathway enrichment analysis considering both differentially expressed genes (enrichment score [ES]) and metabolites (pathway abundance score [PAS]). A score of 1 (red) indicates co enrichment in cancer (ES and PAS >0), and a score of −1 (blue) indicates co enrichment in normal samples (ES and PAS <0). (C) Bar plot displaying numerical values for ES and PAS for the purine catabolism pathway in three different tissues: breast, bladder, and prostate. (D) Heatmap of differentially expressed genes for the purine catabolism pathway in breast cancer. Red cells indicate upregulated genes in TCGA, blue cells indicate downregulated genes in TCGA, and cells marked with an asterisk (∗) correspond to a statistically significant DE (adjusted *p* <0.01). (E) A functional interaction network between genes (ovals) and metabolites (diamonds) in the purine catabolism pathway in breast cancer. Red nodes indicate upregulated components, blue nodes indicate downregulated components, and green nodes indicate no information available. The network shows that over-activation of the pathway is contributed by over-expression of both genes and metabolites. (F) Sunburst chart (left panel) displaying the distribution of co-differentially regulated receptor-enzyme pairs across two different clusters similar to receptor-ligand co-regulation analysis (Figure S7). Darker red represents the number of receptor-enzyme pairs significantly co-up regulated in TCGA (i.e., LFC > 1 and adjusted *p* <0.01) and darker blue represents the number of receptor-enzyme pairs co-downregulated in TCGA (i.e., LFC < 1 and adjusted *p* <0.01). Paler colors represent the number of pairs wherein either the receptor or enzyme is significantly differentially expressed (i.e., |LFC| > 1 for both but adjusted *p* <0.01 for only one of these). Gray indicates pairs with anti-regulation or no fold change at all in at least one of them. Only those pairs that are affected in at least 25% of TCGA subtypes are utilized for the clustering. For each cluster, bar plots (center and right) indicate G-protein coupling preferences as well as GPCR families represented as count of the number of receptors.

We found, on average, 63 and eight significantly regulated enzymes and biosynthetic pathways, respectively, across cancers, suggesting a widespread dysregulation of GPCR-ligand biosynthetic pathways in human malignancies, which in some cases was specific to distinct cancer subtypes (Figure 3A). Clustering of biosynthetic pathways via enrichment scores revealed two main groups: one characterized by processes, such as those related to selenocysteine synthesis and di/triphosphate nucleotide interconversion, which are overall upregulated in most cancer subtypes; and one instead characterized by processes recurrently downregulated, such as phase I, functionalization of compounds, or eicosanoids (Figure 3A). Other biosynthetic pathways are specifically enriched in given cancer subtypes. For example, arachidonic acid metabolism is downregulated in several kidney and skin cancer subtypes, while it is significantly upregulated in ovary mesenchymal and pancreas glandular (GL) subtypes. Other pancreatic cancer subtypes, such as the transitional (TR) and undifferentiated (UN),^24^ displayed instead significant enrichment of the synthesis of prostaglandins (PGs) and thromboxanes (TXs) pathway (Figure 3A).

In order to determine if differentially expressed gene transcripts are mirrored by DE of the corresponding metabolites, we compared pathway enrichment scores of differentially expressed transcripts with differential abundance scores of differentially expressed metabolites from a pan-cancer metabolomic profiling dataset,^25^ considering a set of curated biosynthetic pathways (e.g., Reactome; see STAR Methods, and Table S5). We found a total of 33 significant pathways (FDR < 0.01) in at least one of the five cancer types considered, which showed concordant pathway enrichment when considering both differentially expressed transcripts and metabolites (Figure 3B). The cancer type showing the highest number of concordant signatures is pancreatic adenocarcinoma (PAAD). Certain pathways show concordant upregulation in multiple cancer types, including activation of gene expression by SREBF (SREBP), purine catabolism, and pyruvate metabolism, each found upregulated in three distinct cancer types (Figure 3B). For example, purine catabolism is always upregulated when considering both transcriptome and metabolome in breast, pancreas, and prostate cancers (Figure 3C). Inspection of differentially expressed genes (Figure 3D) and metabolites on a functional interaction network shows that the over-activation of the pathway is contributed by the over-expression of both transcript and metabolite components (Figure 3E). However, only a few enzymes or metabolites in this pathway are invariably over-expressed across cancers (e.g., *NUDT1*, *LEF1*, 9H-xanthine; Figures 3D, and S6). Other processes show concordant enrichment with a cancer dependent directionality; e.g., nicotinate metabolism, which is upregulated in pancreas and downregulated in prostate, or creatine metabolism, downregulated in pancreas and upregulated in breast (Figure 3B). Several other pathways are exclusively enriched in specific cancer tissues; for example, glucagon-like peptide-1 (GLP1) regulates insulin secretion, synthesis of PGs and TXs, and acetylcholine regulates insulin secretion are hallmarks of pancreatic cancer and might contribute to reinforcing the known role of the Gs-PKA signaling axis in this cancer type.^26^^,^^27^

Similarly to receptor-ligand pairs, we correlated changes in expression for receptor-enzyme pairs. Also in this case, we found two major clusters, characterized by a majority of concordantly up- (i.e., cluster 1) or downregulated (i.e., cluster 2) enzyme-receptor pairs (Figures 3F; and S7). In the first cluster we found exclusive upregulation of axes including those of 5-hydroxytryptamine and adenosine receptors with biosynthetic enzymes for their cognate ligands (Figures 3F, and S7). In the second cluster, we found exclusive concordantly downregulation of prostanoid and calcium-sensing receptors (Figures 3F; and S7). Other receptor classes are represented in both clusters, via the involvement of distinct receptors and enzymes members (e.g., adrenoceptors, metabotropic glutamate receptors). On average, we found 63 receptor-enzyme pairs whose expression is significantly correlated with respect to a background random model (Figure S8, and Table S6; see STAR Methods).

### Expression of GPCRs signaling network components correlates with patient survival

We systematically assessed correlations between expression of GPCR network components and patient survival. Briefly, we defined groups of patients based on the expression of a GPCR component, i.e., high or low expression, respectively, if above or below the median of the distribution of expression values of that gene in the samples and tested whether these groups had significantly different survivals (see STAR Methods).

**Figure 4:**
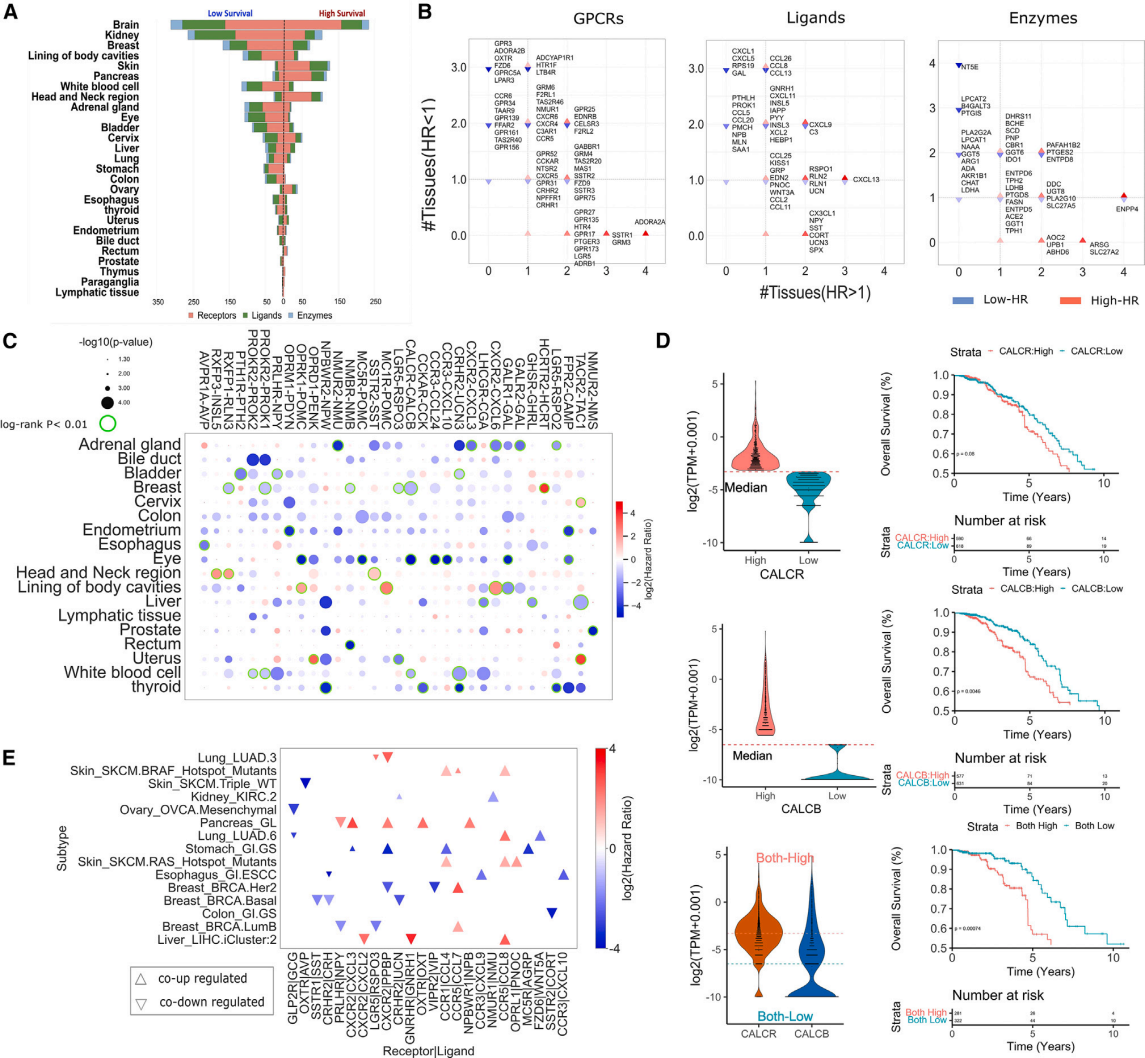
Association of GPCR-peptide ligands axes to survival (A) Survival association of GPCR components in various cancer types. The funnel plot shows the number of significant instances of individual components, such as receptors, ligands, and enzymes, whose expression values are associated with patient survival across various cancer types. A total of 302 unique significant receptor instances (log rank *p* <0.05, FDR < 0.1) were identified in 11 cancer tissues, 103 ligands in 10 cancers, and 71 enzymes in 10 cancers. (B) Scatterplots for individual receptor instances, enzymes, and ligands associated with patient survival in various cancer types. (log rank *p* <0.05, FDR < 0.1). The x axis displays the number of tissues in which the genes are associated with lower survival (inverted blue triangles) and, vice versa, y axis displays the number of tissues in which the genes are associated with higher survival (red triangles). Only the genes that are significantly associated with survival in at least two tissues are shown. (C) The bubble plot shows the correlation between the combined expression levels of GPCR-ligand pairs and patient survival across various cancer types. The pairs with a log rank *p* < 0.05 and also lower than log rank *p* values for individual GPCR/ligand are displayed. Bubble color is proportional to HR; i.e., HR > 1, high expression is correlated with high survival (red); HR < 1, high expression is correlated with poor survival (blue). Bubble diameters are proportional to the −log10(log rank *p* value). Green highlighted bubbles represent the most significant instances (sample sizes >5, FDR < 0.1). (D) The expression values for the CALCR-CALCB axes in breast cancer (right) and corresponding Kaplan-Meier (KM) curve for survival analysis of patients stratified based on the individual as well as combined receptor/ligand expression. (E) Scatterplot showing with differentially co-expressed GPCR-ligand pairs (upper triangle, co-upregulation; lower triangle, co-downregulation) that were significantly associated with patient survival (red, higher survival; blue, lower survival). A total of 24 receptor-ligand pairs from 15 cancer subtypes were identified. Reported *p* values inside KM plots have been obtained through the log rank test.

We first considered the capability of the expression of individual components (e.g., receptors, transducers, ligand, or biosynthetic enzymes) to discriminate patients based on their survival. We found a total of 302 unique significant receptor instances (log rank *p* value <0.05, FDR < 0.1) in 11 cancer tissues, 103 ligands in 10 cancers, and 71 enzymes in 10 cancers (Figure 4A, and Tables S7–S9). Some cancer tissues display overall more components whose expression is associated with higher (e.g., pancreas, skin, or head and neck) or lower (e.g., brain, breast, lining of body cavities, and white blood cell) survival (Figure 4A). Overall, the expression of GPCR genes is associated with poorer survival in 59% of cases across all cancers. *CELSR3*, *GPR25*, *EDNRB*, and *F2RL2* are significantly associated with patients’ survival in four cancers each, with an equal prevalence for lower and higher survival (Figures 4B, and Table S7). Some receptors show consistent associations across cancers; for example, *ADORA2A* is invariably associated to higher survival across four distinct cancer tissues (including pancreas, breast, skin, and head and neck; Figure 4B), which might be consistent with its known role in regulating CD8^+^ T cell activity and survival in the TME.^28^ Certain receptors are instead invariably associated with poorer survival, such as *OXTR*, *ADORA2B*, *GPR3*, *FZD6*, *GPRC5A*, and *LPAR3*. We also found a large prevalence of associations between GPCR-peptide ligand precursor expression and lower survival of patients, with 67.2% of the significant instances across cancers. Among the most recurring ligands, we found several instances that are either invariably associated to lower survival, e.g., *CXCL5*, *CXCL1*, *GAL*, and *RPS19*, or have opposite associations depending on the cancer type, e.g., *CXCL9* (Figures 4B, and Table S8).

We reasoned that, in those patients with higher expression of both receptors and ligands (or biosynthetic enzymes), a likely enhanced activation of the signaling axis should better correlate with specific patient phenotypes (e.g., lower or higher survival) than when considering individual components. We therefore assessed differences in the survival of patients grouped based on the expression levels of GPCRs and ligand precursors (or enzymes), either alone or in combination. We then shortlisted receptor-ligand pairs characterized by higher significance as well as greater hazard ratios of patient survival compared to the individual interaction partners (Figure 4C, and Tables S10–S11, see STAR Methods). Intriguingly, we found several axes, such as *NPBWR2-NPW* or *CALCR-CALCB*, associated with lower survival more significantly than considering interaction partners alone in multiple cancer types (Figures 4C and 4D, and S9). Certain cancer types show multiple significant instances of associations between receptor-ligand axis and survival (e.g., lower survival in adrenal gland, eye; Figure 4C), suggesting a potential polypharmacology strategy for these tumor types.

Interestingly, it appears that some receptors participating in significant axes within the same cancer type share, to some extent, similar signal transduction mechanisms. For example, in head and neck cancers, we found significant correlation of higher survival and higher expression of the *SSTR2-SST* (Figure S10A), *RXFP3-INSL5*, and *RXFP1-RLN3* signaling axis, which all share similar coupling profiles (e.g., G_q/11_, particularly *GNA14*, as well as G_i/o_; Figure S11). *RXFP1-RLN3* is instead associated with lower survival in breast carcinoma patients, highlighting the specificity of function and survival associations of these axes with cancer subtypes. Likewise, *SSTR2-SST* is associated to lower survival in adrenal gland patients (Figure S10B).

Notably, some of the receptor-ligand pairs that we found differentially co-regulated in terms of expression are also significantly associated with survival differences (Figure 4E). In detail, we found a total of 24 receptor-ligand pairs from 15 cancer subtypes. *CCR5* is the receptor-ligand forming pair (with *CCL7* and *CCL8*) that is more frequently concordantly differentially expressed, being at the same time significantly associated with survival. In particular, *CCR5* concordant upregulation is invariably associated with a better prognosis, irrespective of the cancer subtype considered. Other pairs, such as those mediated by *CXCR2*, display cancer subtype-specific patterns of both DE and correlation with patient survival (Figure 4E). On the other hand, pairs such as *CCR1-CCL4* are always co-upregulated but with different prognostic values; i.e., they are associated to higher survival in melanoma (RAS and BRAF hotspot mutant subtypes), and to lower survival in genomically stable gastric cancer (GI.GS subtype; Figure 4E). The latter is also an example of cancer subtype with multiple axes associated to lower survival. Other subtypes, such as the glandular (GL) pancreas, are instead characterized by multiple axes associated to higher survival (Figure 4E). Overall, we found 124 receptor-ligand pairs in 48 cancer subtypes more significantly correlated to survival than their individual instances (Figure S12).

### Biosynthetic enzymes and cognate GPCRs form surrogate signaling pairs with prognostic value

We found multiple ligand-synthesizing enzymes whose expression correlates with patients’ survival in multiple cancers, with 70% of them significantly linked to worse survival (log rank *p* value <0.05, FDR < 0.1). Remarkably, the 5′-nucleotidase *NT5E* is invariably associated to lower survival in four distinct cancer types, namely breast, brain, stomach, and pancreas (Figure 4B, and Table S9). Other enzymes most recurrently associated with lower survival are lysophosphatidylcholine acyltransferase 2 (*LPCAT2*), beta-1,4-galactosyltransferase 3 (*B4GALT3*), as well as prostacyclin synthase (*PTGIS*). On the other hand, enzymes such as bis(5′-adenosyl)-triphosphatase (*ENPP4*), arylsulfatase G (*ARSG*), and the long-chain fatty acid transport protein 2 *(SLC27A2*) are recurrently associated to higher survival (Figure 4B).

**Figure 5:**
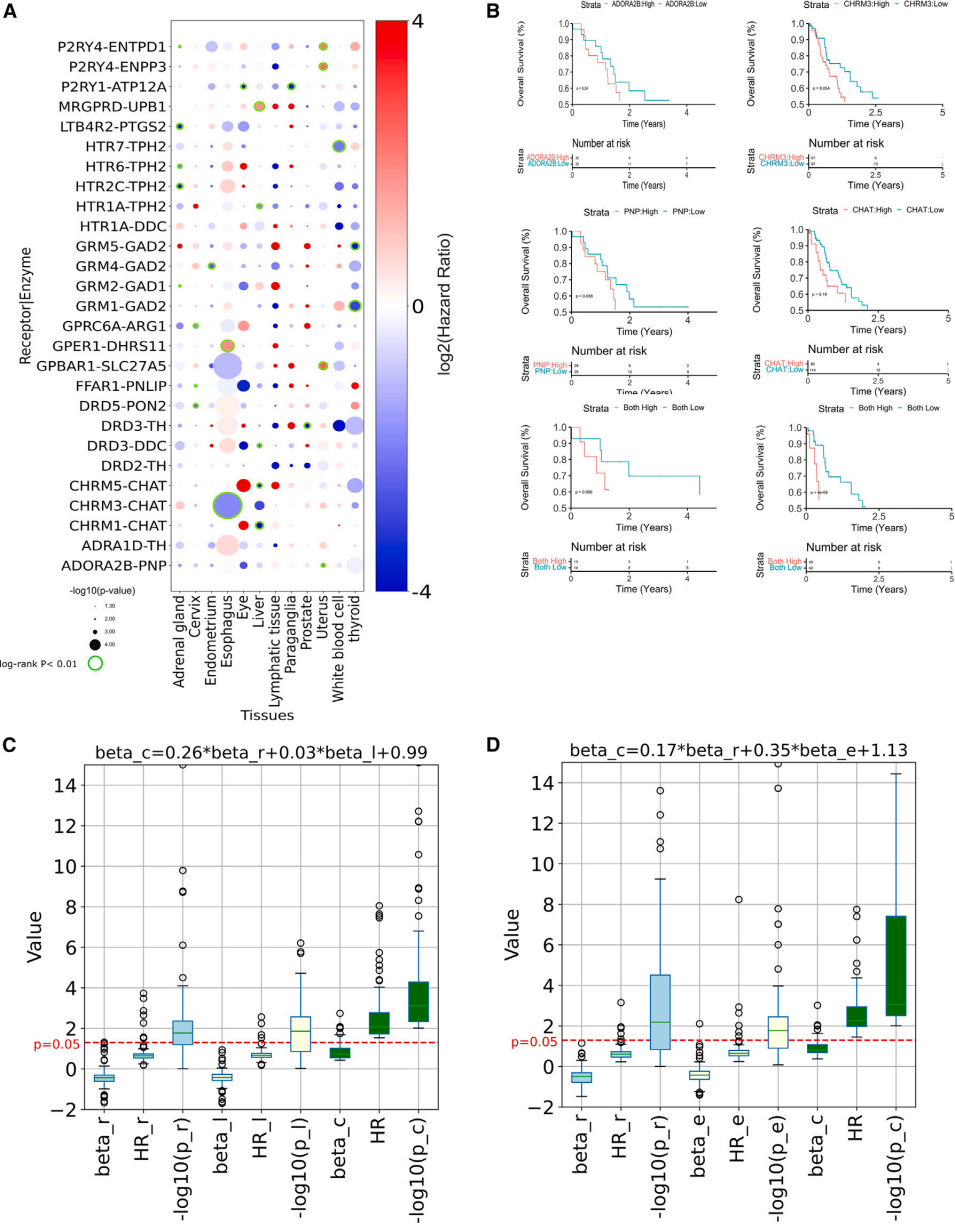
Association of GPCR-enzymes axes to survival (A) The bubble plot shows the correlation between the combined expression levels of GPCR-enzyme pairs and patient survival across various cancer types. The pairs with a log rank *p* < 0.05 and also lower than log rank *p* values for individual GPCR/enzyme are displayed. Further, the pairs with rate-limiting enzymes having less than 100 PubMed references are not shown. Bubble color is proportional to HR; i.e., HR > 1, high expression is correlated with high survival (red); HR < 1, high expression is correlated with poor survival (blue). Bubble diameters are proportional to the −log(log rank *p* value). Green highlighted bubbles represent the most significant instances (sample sizes >5, FDR < 0.1). (B) The CHRM3-CHAT axis is recurrently correlated with lower survival rates in esophageal cancer. The KM plots (left) display the risk enhancement achieved when CHRM3-CHAT combined stratification was applied as compared to individual CHRM3/CHAT as evident by log rank *p* values. Similarly, the ADORA2B-PNP axis is also consistently associated with lower survival rates in uterine cancer, as evident from the KM plot on the right. Reported *p* values inside KM plots have been obtained through the log rank test. (C and D) The boxplot visualizes the results of the receptor-ligand (C) and receptor-enzyme (D) combinatorial Cox proportional hazards (PH) analysis. Each of the nine boxes on the x axis represents distinct parameters, including Cox PH regression coefficients for individual receptor (β_r), ligand (β_l), or enzyme (β_e), and their combinatorial effect (β_c). Additionally, hazard ratios (HRs) and −log10(*p* values) for each component are depicted. The red horizontal line at 1.3 serves as the −log10 transformed significance cutoff (log rank *p* < 0.05) for model selection. The multiple linear regression equation (β_c = f(β_r, β_l, or β_e)) above the plot reflects the relationship between individual and combinatorial regression coefficients. Medians and error bars provide a comprehensive overview of the statistical significance and impact on survival across various cancer datasets.

We similarly tested whether improvement of the survival statistics could be achieved when considering the combined expression of receptor-enzyme interaction partners (STAR Methods; Table S11). This revealed several instances involving biosynthetic enzymes for neurotransmitters and cognate receptors (Figure 5A, and Table S11). Multiple pairs involve enzymes for monoamine neurotransmitter biosynthesis, such as tryptophan 5-hydroxylase 2 (*TPH2*), the rate-limiting enzyme for serotonin synthesis, which is significantly associated to lower survival with multiple serotonergic receptors (e.g., *HTR1A* in liver, *HTR2C* and *HTR6* in adrenal gland, and *HTR7* in white blood cell). Several dopamine receptors are significantly correlated along with enzymes mediating catecholamine biosynthesis, including *DDR3-DDC* (liver) and *DDR2* and *DDR3-TH* (prostate). The latter enzyme, which rate limits the catecholamine pathway, is also significantly associated with *ADRA1D* in lymphatic tissue. The axes formed by choline O-acetyltransferase (*CHAT*) and muscarinic receptors *CHRM1*, *3*, and *5* are associated with lower survival in multiple cancers (Figures 5A and 5B), including liver and esophageal cancer (Figure 5A). We found multiple instances of glutamate receptors (e.g., *GRM1*, *2*, *4*, *5*) and glutamate decarboxylase 1 and 2 (*GAD1*, *2*), with the axes of *GAD2* with *GRM1*, *4* and *5* being always significantly associated with lower survival in endometrium and thyroid cancer tissues. We also found several instances of receptors for purines whose aberrant expression co-occur with that of multiple enzymes of the purine catabolism pathway. In detail, while the *ADORA2B-PNP* pair is associated to lower survival in uterus (Figures 5A, and 5B), *P2RY4* axes with *ENPP3* and *ENTPD1* are associated to higher survival in the same cancer. The latter axis is instead significantly associated with lower survival in adrenal gland (Figure 5A). Overall, we found 115 receptor-enzyme pairs in 45 cancer subtypes more significantly correlated to survival than individual instances (Figure S13).

To begin addressing the different contribution of the axis components to the survival analysis, we performed univariate Cox PH to assess the impact of individual receptor and ligand/enzyme pairs on survival, deriving cancer subtype-specific Cox regression coefficients. Subsequently, we conducted combinatorial Cox PH analyses for receptor-ligand and receptor-enzyme pairs from our network, formulating predictor variables based on these coefficients and evaluating their combined effects on survival across diverse cancer types. We then employed multivariate linear regression to scrutinize the relationship between combinatorial and individual receptor, ligand, or enzyme regression coefficients for selected models (see STAR Methods). We found greater coefficients for receptors-enzymes than for receptors-ligands, indicating a higher prognostic impact of the former (Figures 5C and 5D), which is likely due to the complex regulatory networks of biosynthetic enzymes.

### Drugs for GPCR networks hold the potential to inhibit cancer cell growth

**Figure 6:**
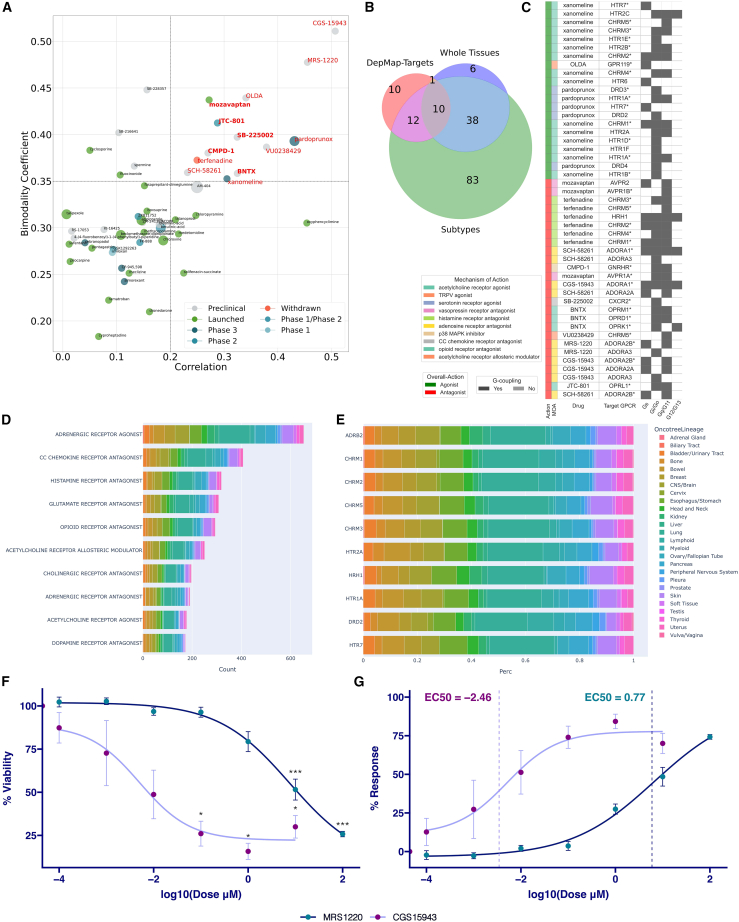
GPCR ligands with cancer cell line growth-inhibitory capacity (A) The scatterplot displays 52 GPCR ligands that were tested in 578 cell lines in PRISM drug repurposing resource. Thirteen GPCR ligands (in red) were found to significantly inhibit the growth of cancer cell lines (correlation > 0.2, bimodality coefficient >0.35). (B) Venn diagram showing the overlap of GPCRs in the target proteins of these 13 ligands with the pooled list of GPCRs that were found to mediate survival-associated GPCR-ligand/GPCR-enzyme axes. (C) The heatmap displays the 13 shortlisted ligands and their corresponding GPCR targets. The targets marked with asterisks (∗) are the GPCRs overlapping with significant axes. Heatmap also displays the overall action mode of ligand, more detailed mechanism of action (MOA), and G-protein coupling associated with the GPCR. (D) Stacked bar plot of top 10 MOAs involving the top 10 GPCR drug with the highest growth inhibition potential (ranked according to the negative of the log2FC in each cell line). Each stack is proportional to the number of cell lines of a given tissue where a drug belonging to that MOA is ranked among the top 10 inhibiting drugs. Stack coloring is tissue specific. (E) Stacked bar plot, normalized for the total cell line-drug pairs count, of top 10 drug targets hit by the top 10 GPCR drug with the highest growth inhibition potential (ranked according to the negative of the log2FC in each cell line). Each stack is proportional to the number of cell lines of a given tissue where a drug hitting the specific target is ranked among the top 10 inhibiting drugs. Stack coloring is tissue specific. (F) Graph showing viability changes in HEPG2 cells following treatment with MRS1220 and CGS15943, normalized to non-treated cells. To assess the statistical significance of the difference in the mean of the non-treated and treated cells, pairwise Wilcoxon test was used (non-significant, *p* > 0.05; ∗*p* ≤ 0.05; ∗∗*p* ≤ 0.01; ∗∗∗*p* ≤ 0.001). (G) Dose-response curve depicts the corresponding response of the HEPG2 cell line to the drugs and the calculated log10 of EC_50_. The error bars indicate the standard error of the mean.

We retrieved GPCRs ligands showing cancer-growth-inhibitory capacity from a public resource containing the activity of 4,518 drugs tested across 578 human cancer cell lines.^29^ A total of 13 out of 52 GPCR ligands tested were found to significantly inhibit the growth of cancer cell lines (Figure 6A).^29^ We checked the targets of these ligands on the pooled list of GPCRs that we found to mediate axes significantly associated with cancer survival. Strikingly, all these drugs bind to receptors that are significantly co-regulated axes with either their ligands or biosynthetic enzymes (Figures 6A, 6B, and Table S12). In particular, among them we found multiple ligands targeting certain GPCRs classes, including adenosine receptors (i.e., CGS-15943, MRS-1220, SCH-58261), muscarinic receptors (i.e., VU0238429, xanomeline, terfenadine), and opioid receptors (i.e., BNTX and JTC-801). Interestingly, the vast majority (76%) of these drugs are antagonists. Moreover, receptors targeted by antagonists appear to be more promiscuous and coupled primarily to G_q/11_ or G_i/o_ but also to a lesser extent to G_s_ and G_12/13_ proteins (Figure 6C).

We also considered an updated version of the PRISM (profiling relative inhibition simultaneously in mixtures) assay of cancer cell sensitivity by pooling the data of 1,514 GPCR drugs tested against 906 cell lines (Figures 6D and 6E; see STAR Methods). The GPCR drugs with the highest potential of cancer cell line growth inhibition target adrenergic receptors, CC chemokine, glutamate, histamine, opioid, acetylcholine, and dopamine receptors (Figure 6D). Among the top 10 individual targets are *ADRB2*, muscarinic, 5-hydroxytryptamine, and *DRD2* receptors (Figure 6E). While cell lines from certain tissues seem to be equally inhibited by top GPCR drugs (e.g., lung), others are sensitive to specific GPCR drugs, such as lymphoid cancer cells, which are particularly inhibited by *DRD2*, *CHRM1*, *CHRM2*, *HTR1A*, and *HTR2A* antagonists (Figure 6E, and Table S13). Multiple targeted GPCRs are expressed across cell lines. At the transcriptomics level, *ADORA2B* is the single most expressed GPCR across cell lines (Figure S14A), while, at the proteomic level, HRH1 is the most expressed (Figure S14B).

We have validated the growth-inhibitory potential of two of the most promising GPCR drug candidates. Given the increasing evidence showing anti-tumor effects of adenosine receptor targeting in hepatocellular carcinoma (e.g., Allard et al., Mazziottaet al., Myojin et al.^30^^,^^31^^,^^32^), we focused our analysis on liver cancer cell lines (Figure S14C), which we used to test adenosine receptor inhibitors. In detail, we performed cell-line drug response and viability assays on the human HEPG2 hepatoblastoma cell line for CGS-15943, an *ADORA2A* and *ADORA1* inhibitor, which is reported to act against multiple adenosine receptors (Figure 6C), and MRS-1220, an *ADORA3* and *ADORA2B* inhibitor. We showed that the viability of the HEPG2 cell lines is significantly reduced upon both CGS-15943 and MRS-1220 treatment (Figures 6F, and S15) in a dose-dependent manner, with a half-maximal effective concentration (EC_50_) of 0.003 μM and 5.9 μM, respectively (Figure 6G), which is very close to or falls within the EC_50_ range of the liver cell lines tested with the same drugs in PRISM (0.009–3.8 μM and 0.01–12 μM; Figure S15C).

## Discussion

In recent years, the multifaceted role of GPCR signaling in cancer has begun to emerge,^15^^,^^16^ although an overall mechanistic picture is still lagging. At a molecular level, this is mainly due to the lack of a precise mechanistic understanding of how receptors transduce external signals intracellularly in the context of the TME. Additionally, there is a lack of clarity regarding how the upstream ligand and biosynthetic pathway components contribute to trigger an aberrant GPCR signaling network in tumor cells as well as in their associated stroma and infiltrating immune cells. At a higher level, the complex circuitry wired by multi-component GPCRs systems is highly tailored to specific cellular contexts. Pathways dysregulation might be initiated through perturbations of these multi-component systems at different levels, all of which may converge to an overall dysregulation and/or persistent activation of GPCR downstream signaling.

In the present study, we systematically inferred GPCR pathways activities from cancer transcriptomic datasets, with a special focus on the dysregulation of GPCR signaling axes involving the co-regulation of the expression of their direct ligands as well as biosynthetic pathways controlling ligand availability. We showed that GPCRs activity is associated to cancer-specific pathways and transcriptional programs. We found subtype-specific patterns of transcriptional regulators that are significantly over-represented for differentially expressed GPCRs. Notably, the transcriptional regulator most associated with GPCR deregulation is *HMGA1*, a chromatin protein that was reported to promote cancer aggressiveness in part via Wnt signaling amplification.^33^ Another recurrently associated transcriptional regulator is *TEAD1*, a transcription factor that recruits the YAP/TAZ coregulators to promote cancer cell proliferation, survival, and stemness.^34^ We found that multiple GPCRs are co-regulated together with their cognate ligands or biosynthetic enzymes, suggesting a coordinated effect on the signaling axis. Some of these are widespread and co-regulated across cancers; others are affected in specific tumor contexts. This study revealed some emerging patterns, among them that chemokine receptors and their cognate chemokines are certainly the most co-regulated axes. The precise role in pro- or anti-tumor immunity played by these chemokine receptor networks is known to depend on the precise cellular and tumor subtype context.^15^^,^^35^ We have reported *CXCR3* to be concordantly upregulated together with its ligands across cancer subtypes. Intriguingly, its expression in regulatory T (Treg) cells has been recently shown to be central for immune suppression of the CD8^+^-mediated response in multiple cancers.^36^ Our map also revealed slightly different co-regulation patterns for chemokine axes entailing different types of ligand action on the receptors, such as the known *CXCR3* agonist *CXCL11*, which also antagonizes *CCR3*.^37^ The known tumor-promoting *CXCR2* is found co-downregulated together with *CXCL2* in liver cancer (iCluster2 subtype) and with *PPBP*, the precursor of *CXCL7*, in lung (LUAD.3 subtype). Remarkably, in both cases, the co-downregulation is associated with higher survival (Figure 4E). The co-upregulation of this axis is associated with lower survival in stomach cancer (genomically stable GI.GS subtype) and with higher survival in pancreatic cancer (GL subtype), similarly to *CXCR2*-*CXCL3*. Other chemokine receptors, such as *CCR1*, *CCR3*, or *CCR5*, are significantly co-upregulated across different cancer subtypes. While *CCR1-CCL4* has distinct correlation to survival depending on the cancer type considered, *CCR5-CCL7* or *CCR5-CCL8* co-upregulation is always associated with higher survival, suggesting a general anti-tumor immunity of these axes in the specific cancer subtypes (Figure 4E).

Interestingly, we also found a few non-chemokine receptors that are broadly co-up activated, such as *KISS1*, *NPBWR*, *MCHR1*, and *APLNR*, which suggest the existence of signaling mechanisms other than chemokine sustaining the cell-cell interaction within the TME. Another notable example of highly context-specific co-regulation of signaling axes is the one mediated by *CALCR* and its close paralog, *CALCRL*, whose interaction with *ADM* is a critical factor determining relapse and drug resistance in leukemia.^38^ We found that many of the co-regulated signaling axes are also associated with patient survival. For example, in head and neck cancer, we have found multiple signaling axes significantly associated to higher survival, such as *SSTR2-SST*, *RXFP3-INSL5*, and *RXFP1-RLN3*. Intriguingly, all the three receptors show converging couplings toward G_q/11_ and G_i/o_ proteins. Notably, *SSTR2-SST* has been significantly associated to higher survival rates in Epstein-Barr virus (EBV)-nasopharyngeal cancer, a class of head and neck cancer, and its targeting with a peptide-drug conjugate (i.e., PEN-221) has been proposed to treat this type of tumor.^39^ *SSTR2-SST* is also one of the axes with the strongest literature support for a role in cancer, along with several others, including *MC1R-POMC* and *GHRHR-GHRH* (Figure S16, and Table S14).

We found that *GNAS* is widespread over-expressed across cancer (Figures S1B, and S1C), which might be connected to its critical role in controlling cell energetics and metabolism via cyclic AMP (cAMP) and PKA signaling, ultimately sustaining tumorigenesis and resistance mechanisms.^14^^,^^15^ However, many G_s_ receptor-peptide axes (Figure S5), or the whole G_s_ pathway (Figure S1D), are downregulated in many tumor subtypes. We speculate that *GNAS* over-activation, via over-expression, amplification, or activating mutations, would make the over-expression of upstream, cognate receptors most often unnecessary, therefore rendering its activation independent of external stimulus and coupling to cognate receptors, which we recently showed to be a tightly regulated structural process.^11^

Many GPCR axes are de-regulated along with recurrent cancer gene mutations. This is particularly evident for well-known TSGs such as *APC*, *FBXW7*, *ZFHX3*, and *SMAD4*, which are all associated with a prevalence of upregulated signaling axes, or *PIK3R1* and *ATRX*, associated with downregulated GPCRs. While the connection with GPCR signaling is better established for genes such as *APC*, via Wnt signaling,^40^ or *SMAD4*, via transforming growth factor (TGF)-β,^41^ emerging evidence suggests that also *ZFHX3*^42^ and *ATRX*^43^ might control GPCR signaling.

Along with the mRNA expression of peptide ligand precursors, we also considered the expression of synthesizing enzymes as a proxy to model upstream signals from small organic ligands. Indeed, we found a significant correlation of patients’ survival with expression of axes formed by receptors and biosynthetic enzyme pairs. Similarly to the receptor-ligand axes, we found receptor-enzymatic axes recurrently associated with survival in a cancer-type-specific fashion. For example, we found multiple instances of muscarinic receptors and *CHAT* co-regulation with patient outcome. Cholinergic signaling has been established as a key factor mediating gastric cancer tumorigenesis via an NGF-promoted feedforward loop.^20^ Intriguingly, the association between the expression of muscarinic receptors-*CHAT* axis with lower survival in hepatocellular carcinoma correlates with the known involvement of acetylcholinesterase, which opposes *CHAT* function by degrading acetylcholine, and is associated to better prognosis in HCC.^44^ Other biosynthetic enzyme axes that we have identified are also supported by additional lines of evidence from the literature (Figure S16D) and strongly emphasize the role of the signaling of several neurotransmitters in fostering oncogenic growth. The information about biosynthetic enzymes is indeed particularly important for receptor systems such as GPCRs, which frequently bind to organic ligands, and might represent a resource complementing existing knowledge bases for receptor-ligand interactions used to characterize cell-cell communication from spatial transcriptomics datasets.^23^ It will be critical in the future to dissect the cell-context-specific role played by these GPCRs signaling axes by analyzing single-cell as well as spatial transcriptomics RNA sequencing (RNA-seq) datasets, as we recently began exploring in the context of CD8-T cell function.^45^

Taken together, we have found a few hundreds of receptor pairs with either ligands or enzymes to be significantly associated to different survival in tens of cancer molecular subtypes, suggesting a host of actionable opportunities to modulate oncogenic phenotypes. Strikingly, all the GPCR drugs that have been reported to significantly hamper cancer cell line growth in a recent systematic screen^29^ indeed target receptors of axes that we found significantly correlated with patient survival, with a particular emphasis on receptors for neurotransmitters and adenosine. We have independently validated two adenosine receptor inhibitors (i.e., CGS-15943 and MRS-1220) that showed significant capacity to hamper a hepatoblastoma cell line growth. In the future, it will be interesting to explore the anticancer activity of the many GPCR axes highlighted in our analysis, with a particular focus on (biased) agonists, in addition to antagonists, as well as on drug combinations. In addition to directly hampering cancer growth, the targeting of GPCR networks is emerging as a promising therapeutic option in combination with immunotherapy.^15^ For instance, adenosine signaling is an established factor that dampens the immune response in inflamed tissues and targeting of either adenosine receptors (particularly *ADORA2A*) or adenosine-synthesizing enzymes (*NT5E* and *ENTPD1*) is currently being evaluated in combination with cancer immunotherapies.^46^^,^^47^ Moreover, the G_s_-PKA axis has been recently identified as a key pathway dampening the anti-tumor CD8 T cell activity and resulting in immune checkpoint blockade failure. This study suggests that additional G_s_-coupled receptors (e.g., EP_2_, EP_4_, β_1_AR, and β_2_AR), other than *ADORA2A*, could be targeted in combination with immunotherapy.^45^

Ultimately, our data-driven approach, which integrates high-dimensional transcriptomics datasets and highly curated signaling networks, may enable the functional interpretation of GPCRs’ dysregulation mechanisms and guide the development of novel pharmacological strategies and drug repurposing opportunities targeting GPCRs in cancer.

### Limitations of the study

The current analysis of GPCR signaling axes is solely based on DE analysis of bulk RNA-seq data. The integration of proteomics data, of particular value for transmembrane receptors, will help to further validate the expression levels of GPCRs axes components. The integration of untargeted metabolomics data with the transcriptomics and proteomics ones will provide unprecedented insights on the correlations between receptors and cognate small organic ligands. A systematic analysis of GPCR signaling axes on single-cell RNA-seq (scRNA-seq) and spatial transcriptomics datasets will certainly illuminate the role of these receptor systems in mediating TME interaction. The current network only considers known ligand and biosynthetic pathways for non-olfactory receptor (ORs). Given the increasing availability of OR ligands knowledge, it will be possible to integrate the information of biosynthetic pathways and enzymes for specific OR ligands.

## STAR★Methods

### Key resources table

**Table:** 

REAGENT or RESOURCE	SOURCE	IDENTIFIER
**Chemicals, peptides, and recombinant proteins**		

MRS 1220	Tocris Bioscience	Cat#1217; Cas: 183721-15-5
CGS 15943	Cayman Chemical Company	Cat#CAYM22073-5; Cas:104615-18-1
Dimethyl sulfoxide	Sigma Aldrich	Cat#D2438-50ML

**Critical commercial assays**		

Calcein, AM, cell-permeant dye	Life Technologies	Cat#C1430

**Deposited data**		

GPCR-Ligand pairs	Harding et al.^48^	IUPHAR: https://www.guidetopharmacology.org/download.jsp
Enzyme list	Lombardot et al.^49^	RheaDB: https://www.rhea-db.org/help/download
GPCR-Ligand-Enzyme network	This paper	Deposited to SIGNOR: https://signor.uniroma2.it
Reactome pathways	Jassal et al.^50^	https://reactome.org/download-data
RNA-seq count and TPM dataset: TCGA TARGET GTEX	Goldman et al.^51^	UCSC Xena: https://xenabrowser.net/datapages/
TCGA firehose legacy mutation data	Cbioportal datasets	https://www.cbioportal.org/datasets
OncoKB cancer gene list	OncoKB website	https://www.oncokb.org/cancer-genes
PRISM Repurposing 19Q3 Primary Screen	Corsello et al.^29^	DepMap portal: https://depmap.org/portal/download/all/
PRISM Repurposing screens: Repurposing-1M and Repurposing-300	Corsello et al.^29^	DepMap portal: https://depmap.org/portal/download/all/?release=PRISM+Repurposing+Public+23Q2&file=Repurposing_Public_23Q2_Extended_Primary_Data_Matrix.csv
PRISM 23Q4 RNA-seq TPM	Corsello et al.^29^	DepMap portal: https://depmap.org/portal/download/all/?releasename=DepMap+Public+23Q4&filename=OmicsExpressionProteinCodingGenesTPMLogp1.csv
PRISM 23Q4 normalized protein expression	Corsello et al.^29^	DepMap portal: https://depmap.org/portal/download/all/?releasename=Proteomics&filename=protein_quant_current_normalized.csv

**Experimental models: Cell lines**		

Human: HEPG2 cells	Laura Poliseno’s research group (CNR Institute of Clinical Physiology, Pisa, Italy)	RRID:CVCL_0027

**Software and algorithms**		

R TCGAbiolinks	Colaprico et al.^52^	https://github.com/BioinformaticsFMRP/TCGAbiolinks
R caret	NA	https://topepo.github.io/caret/
R tidymodels	NA	https://github.com/tidymodels
R UCSCXenatools	Goldman et al.^51^	https://github.com/ropensci/UCSCXenaTools
R DESeq2	Love et al.^53^	https://bioconductor.org/packages/release/bioc/html/DESeq2.html
R WebGestaltR	Wang et al.^54^	https://cran.r-project.org/web/packages/WebGestaltR/index.html
EnrichR	Kuleshov et al.^55^	https://maayanlab.cloud/Enrichr/
Pan Cancer Metabolism Data Explorer	Reznik et al.^25^	http://projects.sanderlab.org/pancanmet/
MetaboAnalyst	Xia et al.^56^	https://www.metaboanalyst.ca
Cytoscape	Lotia et al.^57^	https://cytoscape.org
R survival 4.3.0	NA	https://cran.r-project.org/web/packages/survival/index.html
R survminer 0.4.9	NA	https://cran.r-project.org/web/packages/survminer/index.html
Custom code	This paper	Github: https://github.com/raimondilab/gpcrsignalingaxes and Zenodo: https://doi.org/10.5281/zenodo.8349771
R ggplot2 3.4.3	NA	https://cran.r-project.org/web/packages/ggplot2/index.html
R drc 3.0.1	NA	https://cran.r-project.org/web/packages/drc/index.html
R ggprism 1.0.4	NA	https://cran.r-project.org/web/packages/ggprism/index.html

**Other**		

Resource webserver complimenting the study	This paper	https://gpcrcanceraxes.bioinfolab.sns.it

### Resource availability

#### Lead contact

For additional details and inquiries regarding the resources, please direct your communication to Francesco Raimondi (francesco.raimondi@sns.it).

#### Materials availability

This study did not generate new unique reagents.

#### Data and code availability

This paper analyzes existing, publicly available data, whose sources are listed in key resources table. All original code has been deposited at Zenodo and is publicly available as of the date of publication. DOIs are listed in the key resources table (https://doi.org/10.5281/zenodo.8349771). The code used for this study as well as for the webapp is freely available via github (https://github.com/raimondilab/gpcrsignalingaxes; https://zenodo.org/records/8349772). Any additional information required to reanalyze the data reported in this paper is available from the lead contact upon request.

### Experimental model and study participant details

For *in vitro* cell culture studies, the human hepatocellular carcinoma cell lines (HEPG2) were generously provided by Laura Poliseno’s research group (CNR Institute of Clinical Physiology, Pisa, Italy). The HEPG2 cell line was isolated from a 15-year-old, male youth with liver cancer. The cells were maintained in Dulbecco’s modified Eagle Medium (DMEM) with L-glutamine (Euroclone, Italy), with 10% fetal bovine serum (Thermo Fisher Scientific, USA), 1% penicillin-streptomycin (Thermo Fisher Scientific, USA). The cells were maintained in a 5% CO2 incubator at 37°C.

### Method details

#### GPCR signaling network

To create an extended GPCR network, we have extracted the GPCR and ligand interactions from the IUPHAR/BPS Guide to PHARMACOLOGY database (IUPHAR database).^48^ We retained only endogenous ligands, thereby extracting 243 GPCRs and 328 ligands. Based on database classification, the considered ligands can be grouped into five groups: 211 peptides, 107 metabolites, 7 synthetic organic, 2 inorganic and 1 natural product. All the other annotations describing GPCRs and ligands were also pulled from the database. To further extend our network we added biosynthetic enzymes for GPCR endogenous ligands. To this end we used Rhea, a curated database of chemical reactions of biological relevance.^49^ To identify metabolizing enzymes for GPCRs ligands, we first referenced IUPHAR ligands to ChEBI,^58^ which is used by Rhea to identify ligands. Ligand mapping between IUPHAR and Rhea was carried out by using the IUPHAR International Chemical Identifiers (InChIs). As many of the chemical reactions are happening under physiological pH of 7.3, the ChEBI identities of participants in Rhea reactions at pH 7.3 were considered. Using our in-house python scripts, we have extracted all the enzymes catalyzing reactions where GPCR ligands participate as products, meaning that most of the reactions have left-right (LR) directionality or, in some cases, opposite (right-left - RL) direction. Moreover, we also considered all the enzymes catalyzing reactions of unknown (UN) direction. Since it is possible that enzyme annotation is available only for parent reactions, i.e., more generic chemical reactions, we also added parent reactions based on Rhea’s hierarchical reaction relationships. For each of the Rhea identities acquired, we extracted the corresponding human enzyme whenever available. We extracted 1492 enzymes linked to GPCR ligands, which were filtered according to the following Gene Ontology cellular components terms: *'plasma membrane', 'axon terminus', 'external side of plasma membrane', 'extracellular', 'neuron projection', 'channel', 'membrane transport'*. We also included in our composite filter additional annotation terms from Pfam,^59^ Panther classification,^60^ Gene ontology processes (GO)^61^ or full UniProt^62^ enzyme name to filter out enzymes classified as kinases, channels, transporters, cytochromes, and adenylyl cyclases. We also included rate-limiting enzymes, by mapping 3k human enzymes from RheaDB onto Reactome pathways from 'Metabolism' domain (R-HSA-1430728), which were first filtered, using similar class and domain annotations as above, to exclude kinases, channels, transporters, cytochromes, and adenylyl cyclases resulting in a total of 2261 unique enzymes, out of which 945 were found to match 163 pathways involving GPCR ligands. To determine which of these enzymes were rate-limiting the whole biosynthetic pathway, we retrieved for each of them the number of PubMed articles using the following search query: '{Enzyme gene symbol} AND (rate limiting OR bottleneck)'. The enzyme with the highest number of PubMed references for each metabolism pathway associated to GPCR ligands was retained (Supplementary Table S15).

After these filtering steps, we yielded a total list of 266 enzymes, each involved in interaction with one or multiple GPCRs mediated by synthesized ligands. Receptor-enzymes were further filtered out by using the STRING database, a knowledgebase of known or predicted protein-protein functional interactions.^63^ Via STRING, we mapped all possible interactions between GPCRs and enzymes producing cognate ligands, by using a minimum confidence score of 150, and leading to a shortlist of 82 enzymes. The final network contained 82 enzymes, 328 ligands and 243 receptors, for a total of 150 binary enzymes-ligand interactions, 646 ligand-GPCR, and 288 receptor-enzyme (ligand-mediated) interactions.

The whole network has been compiled containing different descriptions of each of the three main interactors and their interactions are currently being deposited to SIGNOR^64^ database thanks to an ongoing dedicated curation project.

We created G protein-specific gene sets and annotations by aggregating consensus experimental transducer data (i.e., Universal Coupling Map^9^) as well as predictions through Precogx.^7^

#### Transcriptomics dataset

We considered gene-level expressions from the dataset ‘TCGA TARGET GTEx’ publicly available at UCSC Xena (https://xenabrowser.net/datapages/).^51^ These data were generated using the TOIL pipeline, a portable, open-source workflow software that can be used to run scientific workflows on a large scale in cloud or high-performance computing (HPC) environments.^65^ The Toil pipeline uses STAR^66^ to generate alignments and read coverage graphs, and performs quantification using RSEM^67^ and Kallisto.^68^ It was utilized to process ∼20,000 RNA-seq samples to create a consistent meta-analysis of datasets free of computational batch effects.

We extracted the gene-level expression data, *RSEM expected_count*, using the R package ‘UCSCXenatools’ for 19109 samples. Thereafter, the TARGET samples were filtered and only TCGA and GTEx samples were retained (*n* = 18305; Table S2). Due to unavailability of GTEx data for all the tissue types, only 16 whole cancer-types (TCGA) were contrasted to the healthy samples pertaining to the same tissue (GTEx). The information for 76 distinct TCGA subtypes was retrieved from TCGAbiolinks^52^ (R4.2, Bioconductor v3.16) except for ‘Pancreas’ and ‘Testis’ for whom it was unavailable.

For pancreatic cancer we employed a new classification of TCGA samples using a multi-class classifier already implemented to classify three novel PDAC subtypes (Glandular, GL; Transitional, TR; Undifferentiated, UN) obtained from the transcriptional profiles of multiple morphological distinguishable tumor areas isolated by laser micro-dissection (LMD) in primary PDACs of treatment-naïve patients.^24^ Briefly, a random Forest (RF) classification method was used to build the model setting the number of trees to 500. To avoid overfitting issues, the k-fold cross validation was performed. A selection procedure to maintain only the most informative features was implemented using the recursive feature elimination (rfe) function in caret R package (https://topepo.github.io/caret/). The gene expression and clinical data of the TCGA study cohort were obtained by the GDC database and then the samples were further selected for PDAC diagnosis. Read counts were normalized using DEseq2’s median of ratios. Normalized counts were Log2-transformed and were preprocessed by centering and scaling samples using the Tidymodels R package (https://github.com/tidymodels). PDAC subtypes were predicted in each TCGA sample using the model originated above with the predict function in the randomForest R package setting the parameter type to “class”.

#### DE analysis

We obtained log-transformed gene-level count data (RSEM expected_count) from the UCSCXenatools,^51^ which was subsequently inverse-transformed to raw expected count. TCGA matched normal samples were discarded, and a DE analysis was performed to identify genes with significant expression changes across TCGA-GTEx samples. The DE analysis was conducted using DESeq2,^53^ a widely used pipeline for DE analysis. We considered genes with an |LFC|>1 and an adjusted *p* value (Padj) < 0.01 (Benjamini-Hochberg correction^69^) to be differentially expressed. This process was performed for each of the 16 whole tissue types and 79 subtypes. The contrast for each whole tissue, or subtype, was performed using the corresponding GTEx whole tissue dataset.

#### Co-regulation analysis

To identify co-differentially regulated receptor-ligand pairs, we assigned scores to each of these gene pairs. We considered three major cases: (a) both co-recurrently up/downregulated with significant Padj values (a score of −2/+2), (b) both co-recurrently up/downregulated with at least one of them having a significant Padj value (a score of −1/+1), and (c) no or anti co-differential regulation (a score of 0). This information was displayed in the form of Seaborn clustermap (v0.11.2, Python v3.9.7) with hierarchical clustering performed using the ‘ward’ method of the scipy.cluster library (v1.7.1, Python v3.9.7). On top of this heatmap, G-coupling information (see STAR Methods section GPCR - G Protein coupling data) and ligand’s mechanism of action (from IUPHAR) was annotated as separate heat maps using matploltib library (v3.4.3, Python v3.9.7) This way we identified differentially expressed genes and co-differentially regulated receptor-ligand pairs in a comprehensive and robust manner. The use of DESeq2 and Benjamini-Hochberg correction ensured the statistical significance of the results, and the consideration of multiple tissue types and subtypes allowed for a more nuanced understanding of the data.

The gene-gene correlations were calculated using the *pearsonr* function from the scipy.stats library in Python v3.9.7. Subsequently, each correlation coefficient was compared to a background distribution of correlation coefficients derived from 1000 randomly selected gene pairs within the respective cancer subtype. The comparison was performed using a t test from the scipy.stats library in Python v3.9.7. The t test examines the likelihood that the observed correlation arises from chance variations rather than representing a genuine relationship. A low *p* value resulting from the t test indicates that the correlation coefficient significantly deviates from the expected background distribution. This suggests a potential meaningful association between the genes under investigation.

To account for multiple hypothesis testing, Benjamini-Hochberg correction was applied to the resulting t test *p* values. The *multipletests* function from the statsmodels library in Python v3.9.7 was utilized for this purpose. A gene pair was considered 'correlated' if the false discovery rate (FDR) corrected t test *p* value was less than 0.05, and the absolute value of the correlation coefficient exceeded 0.25.

We calculated "tissue-wise similarity" and “ligand-wise similarity”, to illustrate GPCR-ligand systems across different TCGA subtypes. The *Tissue-wise Similarity* metric measures the similarity in the differential expression profiles of GPCR-ligand pairs across different tissues. The computation involves determining the average pairwise Euclidean distances between the co-differential expression (co-DE) profiles of GPCR-ligand pairs for each tissue. In essence, it provides a quantitative measure of how closely related or distinct the expression patterns of GPCR-ligand pairs are across various tissues. A higher tissue-wise similarity indicates that the GPCR-ligand pairs exhibit similar expression patterns in different tissues, while lower similarity values suggest diverse expression profiles. The *Ligand-wise Similarity* metric assesses the similarity in the differential expression profiles of a GPCR across its ligands. Similar to the tissue-wise similarity, this metric calculates the average pairwise Euclidean distances between the co-DE profiles of ligands associated with a specific GPCR. This metric offers insights into how consistent or variable the expression patterns of different ligands are when acting through a common GPCR. Higher ligand-wise similarity values suggest that the ligands associated with a GPCR exhibit similar expression patterns, while lower values indicate diversity in the differential expression profiles of these ligands.

#### Gene set enrichment analysis

We performed gene set enrichment analysis (GSEA) using the WEB-based GEne SeT AnaLysis Toolkit (WebGestalt).^54^ The GSEA process was automated using the R package WebGestaltR. We chose the 'reactome' pathway database^50^ for enrichment analysis. The results from these analyses were employed to correlate the differentially expressed genes to various reactome pathway gene sets with a positive (enriched in TCGA) or negative enrichment (enriched in GTEx) score with Padj<0.01. We considered GSEA results for downstream analyses by filtering pathways containing the GPCR ligands or enzymes belonging to either Signal transduction (‘signaling by GPCR’:R-HSA-372790) or Metabolism (‘metabolism’:R-HSA-1430728) reactome upper level domains.

#### Transcription factor enrichment

We utilized the differentially expressed GPCR genes (obtained earlier from DeSeq2) for a systematic transcription factor (TF) enrichment analysis using EnrichR (https://maayanlab.cloud/Enrichr/),^55^ which included leveraging the EnrichR API via Python’s ‘requests’ library for TF database access, and ‘seaborn’ for visualization. Employing established approaches such as Transfac and Jaspar position weight matrices (PWMs), the top five significantly enriched TFs (adjusted *p* value <0.05) for each cancer subtype were identified. This analysis reveals the association between TFs and significantly differentially expressed GPCRs.

For visual representation of relationships among differentially expressed GPCR genes and enriched TFs, a *clustermap* was constructed using seaborn. The *'ward'* method from the scipy library was employed for clustering. The resulting heatmap effectively showcases co-occurrence patterns, with filled cells indicating TF enrichment in specific cancer subtypes. The color intensity corresponds to the count of differentially expressed GPCRs associated with the respective TF in each subtype. Additionally, a barplot atop the clustermap provides insight into the count of subtypes in which each TF exhibited enrichment. This comprehensive approach facilitates exploration of the regulatory TFs governing GPCR genes in diverse cancer subtypes.

#### Comparative transcriptome-metabolome analysis

We mapped differentially expressed transcripts and metabolites onto a functional interaction network from Reactome pathways,^70^^,^^71^ which integrates in the same network information from proteins and small organic molecules. We first collected data from the Pan-Cancer Metabolism Data Explorer,^25^ a comprehensive database that contains information about the differential regulation of metabolites associated with cancer aggressiveness. This resource gathered data from over 900 patient samples from five subtypes of cancer: Breast (BRCA), Prostate (PRAD), Pancreas (PAAD), Kidney (KIRC) and Bladder (BLCA). Using this data, we identified the DE regulation of enzyme(s) associated with ligand biosynthesis. For this purpose, we used the list of our curated enzymes to query within this external dataset using an HMDB to ChEBI mapping done via MetaboAnalyst.^56^ Then, for each pathway, the LFC and Padj values of constituent enzymes were retrieved. Subsequently, this information was merged with the Reactome functional interaction network for a ligand-receptor signaling pathway to complete the entire axis. This was achieved by considering only the first neighbors for involved participants. The resultant networks were used to visualize the directional regulation as well as the DE profiles by utilizing Cytoscape.^57^

#### Pathway Abundance Score

We compared the enrichment scores of differentially expressed transcripts with the differential abundance scores of differentially expressed metabolites obtained from the Pan-Cancer Metabolism Data Explorer. To this end, we respectively calculated the GSEA Enrichment Score (ES) and the Pathway Abundance Score (PAS)^25^ by considering only the filtered set of curated biosynthetic pathways from Reactome, specifically for metabolism (R-HSA-1430728). The PAS was calculated by using the formula (I-D)/S, where I represents the number of metabolites (or enzymes) upregulated (LFC>0) in a pathway, D represents the number of metabolites downregulated (LFC<0) in the pathway, and S is the sum of I and D or 1 if I + D = 0. A pathway is considered enriched in cancer if the PAS is positive and negative otherwise (similar to ES). We utilized these two metrics to qualitatively evaluate the concordance of enriched biosynthetic pathways in our findings.

#### Survival analysis

The study used univariate unadjusted Cox-Proportional Hazards (Cox-PH) regression models to compute hazard ratios (HR) for predicting the risks of death between high-risk and low-risk TCGA patient groups based on overall survival time. The survival and censoring information was retrieved using TCGAbiolinks for 28 TCGA tissues and 118 subtypes. The risk groups were stratified based on median values of expression, and Kaplan-Meier (KM) plots were used to compare their survival curves. 'survival' (v4.3.0) and 'survminer' packages (v0.4.9) in R (v4.2) were used for survival analyses, and statistical significance between survival curves was estimated using log rank tests with *p* values less than 0.05 considered significant.

For the individual components (i.e., Receptors/Ligands/Enzymes), patients were stratified in two risk groups based on the median expression value of each component and HR were estimated. Using the ‘low expression’ group as reference for HR calculations, we label the resulting risk groups as ‘High Survival’ (HR > 1) or ‘Low Survival’ (0<HR < 1). We further employed the combined expression levels for GPCR axes components i.e., GPCR-ligand/GPCR-enzyme for risk stratification. The patients were now stratified in the two risk groups utilizing the median cutoffs of both the components for e.g., for a GPCR-Ligand based stratification, patients with expression value, EV > Median_GPCR_ and EV > Median_Ligand_ were placed in one group and the patients with EV < Median_GPCR_ and EV < Median_Ligand_ in another group. We then filtered out those significant GPCR-axis pairs wherein the Hazard-Ratio axis (HR_axis_) was more than 2-fold of the HR in both the individual stratifications (i.e., for above example [HR_axis_/HR_GPCR_ < 0.5 or HR_axis_/HR_GPCR_ > 2] and [HR_axis_/HR_Ligand_ < 0.5 or HR_axis_/HR_Ligand_ > 2]) along with the criteria that the logrank-p_axis_ is smaller than the logrank-p values value of both of the individual stratifications and significant i.e., logrank-p_axis_<0.05. These instances were then displayed on a heatmap created using Seaborn scatterplot created using matplotlib (v3.4.3, Python v3.9.7) with hierarchical clustering performed using the ‘ward’ method of the scipy.cluster library (v1.7.1, Python v3.9.7).

Survival analyses were performed considering whole cancer tissues, as defined in Xena browser, or at the TCGA molecular subtype level.

#### Expanded pan-cancer survival assessment

##### Univariate and combinatorial Cox PH analyses

Univariate Cox-Proportional Hazards (Cox-PH) analyses assessed the individual impact of receptor and ligand/enzyme genes on survival in each cancer dataset. Dataset-specific Cox regression coefficients (*β*_*r*_ and *β*_*l*_ or *β*_*e*_) were derived using the median expression as the stratification cutoff.

For receptor-ligand (and receptor-enzyme) pairs, we formulated predictor variables using dataset-specific regression coefficients as following:

(1) $${Predictor}_{i,d}=\beta_{r,d}\left(i\right)∙receptor\left(i\right)+\beta_{l,d}\left(i\right)∙ligand\left(i\right)$$

where *β*_*r,d*_(i) and *β*_*l,d*_(i) represent the dataset-specific regression coefficients obtained from the univariate Cox-PH analysis for the *i-*th receptor and ligand gene in dataset (*d*). A similar approach was employed for receptor-enzyme pairs.

A subsequent round of univariate Cox-PH analysis, using the estimated predictors, evaluated their combined effects on survival across diverse cancer types.

##### Best-performing combinatorial models

Pooled results from all datasets identified best-performing combinatorial models based on hazard ratios (*HR*_*c*_) and log rank *p* values (*logrank_p_c*). Selection criteria, as earlier, included a hazard ratio (*HR*_*c*_) twice that of individual receptor (*HR*_*r*_) and enzyme or ligand (*HR*_*e*_ or *HR*_*l*_) models, with a log rank *p* value (*logrank_p_c* < 0.05) less than *logrank_p_r* and *logrank_p_l* or *logrank_p_e*.

##### Multivariate linear regression

For selected models, multivariate linear regression analyzed the relationship (*β*_*c*_ = *f*(*β*_*r*_*, β*_*l*_ or *β*_*e*_)) between combinatorial (*β*_*c*_) and individual receptor (*β*_*r*_), ligand, or enzyme regression coefficients (*β*_*l*_ or *β*_*e*_). The estimated regression coefficients provided insights into how the individual receptors/ligands/enzymes contributed to the overall impact on survival across multiple cancer datasets, when restricted to best performing combinatorial models.

##### Subtype-associated mutational co-regulation analysis

In this study, we conducted a systematic analysis of mutation of cancer genes across multiple datasets, focusing on TCGA projects and specifically utilizing data from the Firehose Legacy datasets. The mutation data, obtained from cBioPortal (https://www.cbioportal.org/datasets), comprised ‘_data_mutations.txt’ files. To ensure biological relevance, we processed this genomic information by extracting key details such as mutation status, tumor sample information, and gene classifications.

Our approach involved filtering for oncogenes and tumor suppressor genes based on the OncoKB cancer gene list (https://www.oncokb.org/cancer-genes), refining the dataset to genes of significant biological importance. Subsequently, we associated these mutations with specific cancer subtypes by integrating subtype information from TCGAbiolinks. We standardized Tumor Sample Barcodes to ensure a consistent representation across all datasets.

To discern the landscape of mutated genes linked to various cancer subtypes, we calculated gene frequencies within each subtype and ranked the top 5 mutated genes per subtype. Thereafter, we integrated the subtype specific axes co-regulation information with cancer genes mutational signatures to analyze correlations between the two. The entire process, encompassing data preprocessing and result compilation, was performed in Python leveraging the Pandas library (Python v3.9.7) and the results were rendered using a Seaborn heatmap combined with a Matplotlib (v3.4.3, Python v3.9.7) stacked barplot.

##### Analysis of GPCR drugs on PRISM drug repurposing resource

We retrieved GPCR ligands with cancer growth-inhibitory capacity from a publicly available resource, i.e., PRISM (Profiling Relative Inhibition Simultaneously in Mixtures).^29^ This drug repurposing resource was developed to identify potential drugs for cancer treatment. To predict drug response, PRISM utilizes genomic data from cancer cell lines and patient-derived xenografts, which are inputted into machine learning pipelines. By taking into account inter- and intra-species variability in drug response, PRISM aims to provide personalized treatment options for specific cancer subtypes. The dataset retrieved from PRISM contained information on the growth-inhibitory activity of over 4,500 drugs tested on 578 human cancer cell lines. This data was filtered based on the drugs which had GPCRs as targets and a list of 52 GPCR drugs/ligands was obtained. To identify active drugs for cancer treatment, PRISM uses the bimodality coefficient and correlation as metrics. The bimodality coefficient measures the separation between the two peaks in a drug response distribution, identifying drugs with a bimodal distribution of response across cancer cell lines. The correlation measures the strength of the relationship between predicted and actual drug response in patient-derived xenograft models. Following a similar approach, we used these metrics (correlation>0.2, bimodality coefficient>0.35) to identify GPCR drugs that are active against specific cancer subtypes. The active drugs were then investigated on the basis of GPCRs associated with survival in either of GPCR-Ligand or GPCR-Enzyme axes. The information was further annotated with G-coupling information and ultimately displayed using visual representations such as scatterplots, venn diagram and heatmaps utilizing seaborn (v0.11.2, Python v3.9.7) and matplotlib libraries (v3.4.3, Python v3.9.7).

We also considered data obtained from two recent PRISM Repurposing screens: Repurposing-1M and Repurposing-300. These screens assess the Log2 Fold Change (LFC) in cell counts, deploying the PRISM assay to evaluate 6658 compounds, of which 1514 target GPCRs. The compounds are administered at a dose of 2.5 μM over a 5-day treatment to 906 cancer cell lines. The final LFC values are consolidated in the https://depmap.org/portal/download/all/?release=PRISM+Repurposing+Public+23Q2&file=Repurposing_Public_23Q2_Extended_Primary_Data_Matrix.csv table, constituting a matrix where rows represent individual treatments and columns correspond to the DepMap IDs. For convenience, this matrix also includes the compounds from the original PRISM Repurposing Primary screen. The research also incorporates metadata for the drugs and the cell lines from Repurposing_Public_23Q2_Extended_Primary_Data_Matrix.csv and the Model.csv file, respectively. Using the Model.csv, we leverage DepMapID to stratify cell lines according to tissue type, following the OncoTree ontology. All of this data is freely accessible on the DepMap portal. For each cell line, we considered the top 10 GPCR drugs inhibiting cancer cell growth, i.e., having the most negative log2fold change. We then performed statistic considering the Mechanism of Action (MOA) and Targets associated to each ligand. To monitor GPCR expression on PRISM cell lines, we downloaded log2 transformed TPM RNA-seq data (https://depmap.org/portal/download/all/?releasename=DepMap+Public+23Q4&filename=OmicsExpressionProteinCodingGenesTPMLogp1.csv), as well as normalized protein expression data (https://depmap.org/portal/download/all/?releasename=Proteomics&filename=protein_quant_current_normalized.csv) from DEPMAP.

##### Cell line culture

The human hepatocellular carcinoma cell lines (HEPG2) were generously provided by Laura Poliseno’s research group (CNR Institute of Clinical Physiology, Pisa, Italy). The HEPG2 cell line was maintained in Dulbecco’s modified Eagle Medium (DMEM) with L-glutamine (Euroclone, Italy), with 10% fetal bovine serum (Thermo Fisher Scientific, USA), 1% penicillin-streptomycin (Thermo Fisher Scientific, USA). The cells were maintained in a 5% CO2 incubator at 37°C.

##### Drug treatment

On the day before treatments, cells were harvested, counted, seeded onto 96-well plates at a density of 10^4^ cells per well, and adhered overnight.

For drug treatment, MRS-1220 (Tocris Bioscience, United Kingdom; CAS#183721-15-5) and CGS-15943 (Cayman Chemical Company, USA; CAS#104615-18-1) were prepared in a stock solution of respectively 10mM and 1.7mM DMSO (Merck KGaA, Germany). Serial dilutions using DMEM were performed to achieve a range of concentrations from 0.1nM to 100μM of the drugs. Control wells were treated with an equivalent volume of DMSO used for drug dilution. The treated and control cells were incubated for 72 h at 37°C, 5% CO2 incubator. All conditions were performed in triplicate to ensure reproducibility.

##### Viability test

72h after treatment, Calcein, AM, cell-permeant dye (Thermo Fisher Scientific, USA) assay was conducted in accordance with the manufacturer’s guidelines. The cells were incubated with the dye for 1 h. Subsequently, the fluorescence signal was measured using the Infinite M200 Nano Quant instrument (Tecan, Austria).

Fluorescence intensity values obtained from untreated cells were regarded as representing 100% cell viability. The concentration of the drugs at which 50% cell growth inhibition (EC50) occurred was determined using the drc package (v 3.0.1, R v 4.1.2). The ggplot (v 3.4.3, R v 4.1.2) and ggprism (v 1.0.4, R v 4.1.2) packages were used to draw the plots. Pairwise comparisons were conducted using the Wilcoxon rank-sum test with continuity correction to assess the differences in treated cells compared to the untreated ones. *p* value adjustment for multiple comparisons was performed using the Benjamini-Hochberg (BH) method.

### Quantification and statistical analysis

The statistical analysis in this study was robustly conducted using a variety of software tools and statistical tests. The DE analysis employed log-transformed gene-level count data obtained from UCSCXenatools, with DESeq2 software utilized for analysis. Significance was determined using an Benjamini-Hochberg (BH) correction adjusted *p* value threshold of <0.01 and |LFC|>1. Correlation analysis involved Pearson correlation coefficients computed using the scipy.stats library in Python, with significance assessed using t tests and BH correction for multiple testing (see ‘Method Details:Co-regulation Analysis’). Tissue-wise and ligand-wise similarity metrics, as defined in Method Details ('Co-regulation analysis’), were calculated to assess GPCR-ligand systems, employing custom scripts in Python. GSEA utilized the WebGestalt tool, with Reactome pathway enrichment analysis performed using the reactome pathway database and a BH adjusted Padj threshold 0.01. Transcription factor enrichment analysis was conducted with EnrichR, incorporating Transfac and Jaspar position weight matrices (PWMs) and a BH adjusted Padj<0.05. Survival analysis employed univariate Cox proportional hazards regression and Kaplan-Meier analysis, with statistical significance assessed using log rank tests and a significance threshold of *p* < 0.05. Furthermore, for stratification of patients to conduct survival analysis, median expression values of respective genes were utilized. The KM plots are complemented with risk tables displaying the number of patients in stratified categories. Comparative transcriptome-metabolome analysis, mutation analysis, and drug response prediction were performed using a combination of Python scripts, Pandas library, and various publicly available databases such as cBioPortal, PRISM, and DepMap (see Method Details). The EC50 values, representing the drug concentrations inhibiting 50% of cell growth, were determined using the drc package (v 3.0.1, R v 4.1.2). Wilcoxon rank-sum tests with continuity correction assessed differences between treated and untreated cells, with *p* value adjustment via the Benjamini-Hochberg method for multiple comparisons. All the softwares have been referenced to appropriate sources in the key resources table.

#### Additional resources

The results from this study have been shared openly through a web app, which can be accessed at gpcrcanceraxes.bioinfolab.sns.it.

## Supplemental information

Document S1. Figures S1–S16

Table S1. Related to Figure 1: GPCR ligands in Reactome pathways (metabolism and signal transduction domains)

Table S2. Related to STAR Methods: List of GPCR ligands with cognate receptors and biosynthetic enzymes from RHEADB

Table S3. Related to STAR Methods: Tissue-wise sample distribution in the data retrieved from UCSCXena

Table S4. Related to STAR Methods: Subtype-specific mutated oncodrivers and G-protein coupling co-regulation

Table S5. Related to Figure 3: Reactome pathways enrichment scores and differential abundance scores (pathway abundance scores) calculated from transcripts and metabolite log2FCs

Table S6. Related to STAR Methods: Expression correlation statistics for receptor-enzyme pairs

Table S7. Related to Figure 4: GPCR-based survival analysis; cancer tissue/TCGA molecular subtypes

Table S8. Related to Figure 4: Ligand-based survival analysis; cancer tissue/TCGA molecular subtypes

Table S9. Related to Figure 4: Enzyme-based survival analysis; cancer tissues/TCGA molecular subtypes

Table S10. Related to Figure 4: GPCR-ligand-based survival analysis; cancer tissues/TCGA molecular subtypes

Table S11. Related to Figure 5: GPCR-enzyme-based survival analysis; cancer tissues/TCGA molecular subtypes

Table S12. Related to Figure 6: GPCR ligand activity from PRISM cancer cell-line drug repurposing hub (https://depmap.org/repurposing/)

Table S13. Related to Figure 6: GPCR ligand activity (log2FC) from PRISM Repurposing Public 23Q2

Table S14. Related to STAR Methods: Literature evidence for GPCR axes

Table S15. Related to STAR Methods: Literature support for rate-limiting role of metabolism pathway enzymes

Document S2. Article plus supplemental information
