# Supplementary material for: The landscape of cancer-rewired GPCR signaling axes
Source: Cell Genom. 2024 May 8;4(5):100557. doi: 10.1016/j.xgen.2024.100557 (PMC11099383; doi:10.1016/j.xgen.2024.100557)
Supplement: Document S1. Figures S1–S16 [file mmc1.pdf]

Cell Genomics, Volume *4*

Supplemental information

The landscape of cancer-rewired GPCR signaling axes

Chakit Arora, Marin Matic, Luisa Bisceglia, Pierluigi Di Chiaro, Natalia De Oliveira Rosa, Francesco Carli, Lauren Clubb, Lorenzo Amir Nemati Fard, Giorgos Kargas, Giuseppe R. Diaferia, Ranka Vukotic, Luana Licata, Guanming Wu, Gioacchino Natoli, J. Silvio Gutkind, and Francesco Raimondi

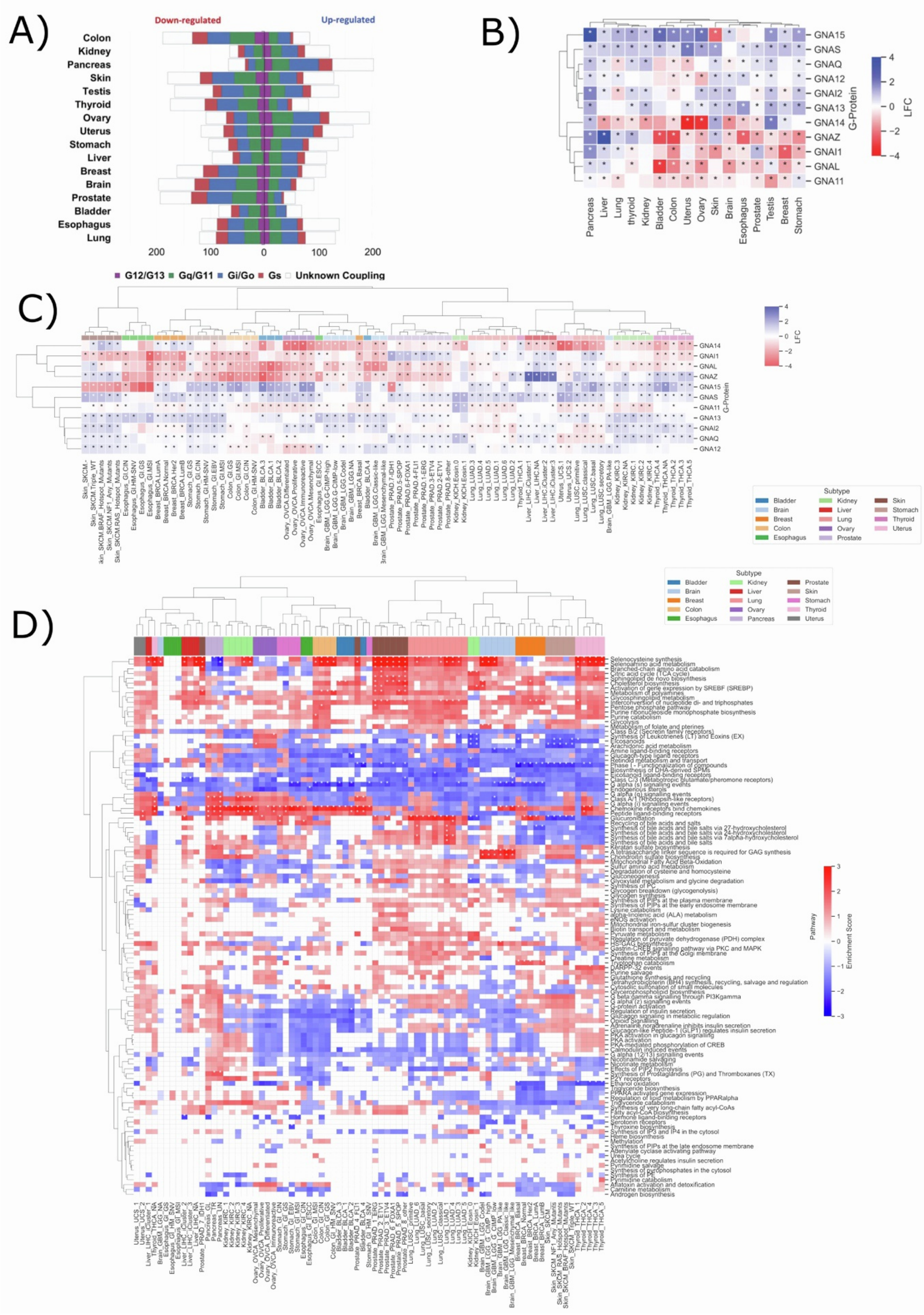

Supplementary Figure S1 GPCR DE analysis and GSEA in cancer tissues, Related to Figure 1 A) stacked barplot with the distribution of DE GPCRs for each cancer tissue categorized by coupling information; B) heatmap showing the LFCs from DE analysis (TCGA vs GTEx, via DESEQ2) of G protein in each cancer tissue. Significant instances (BH Padj<0.01) are annotated with "*"; C) same as B) but for cancer molecular subtypes; D) heatmap showing ligand-associated pathways which are significantly enriched in at least one cancer subtype. An enriched pathway refers to a pathway enriched in TCGA subtype with a GSEA enrichment score>0 and BH corrected Padj<0.01 .

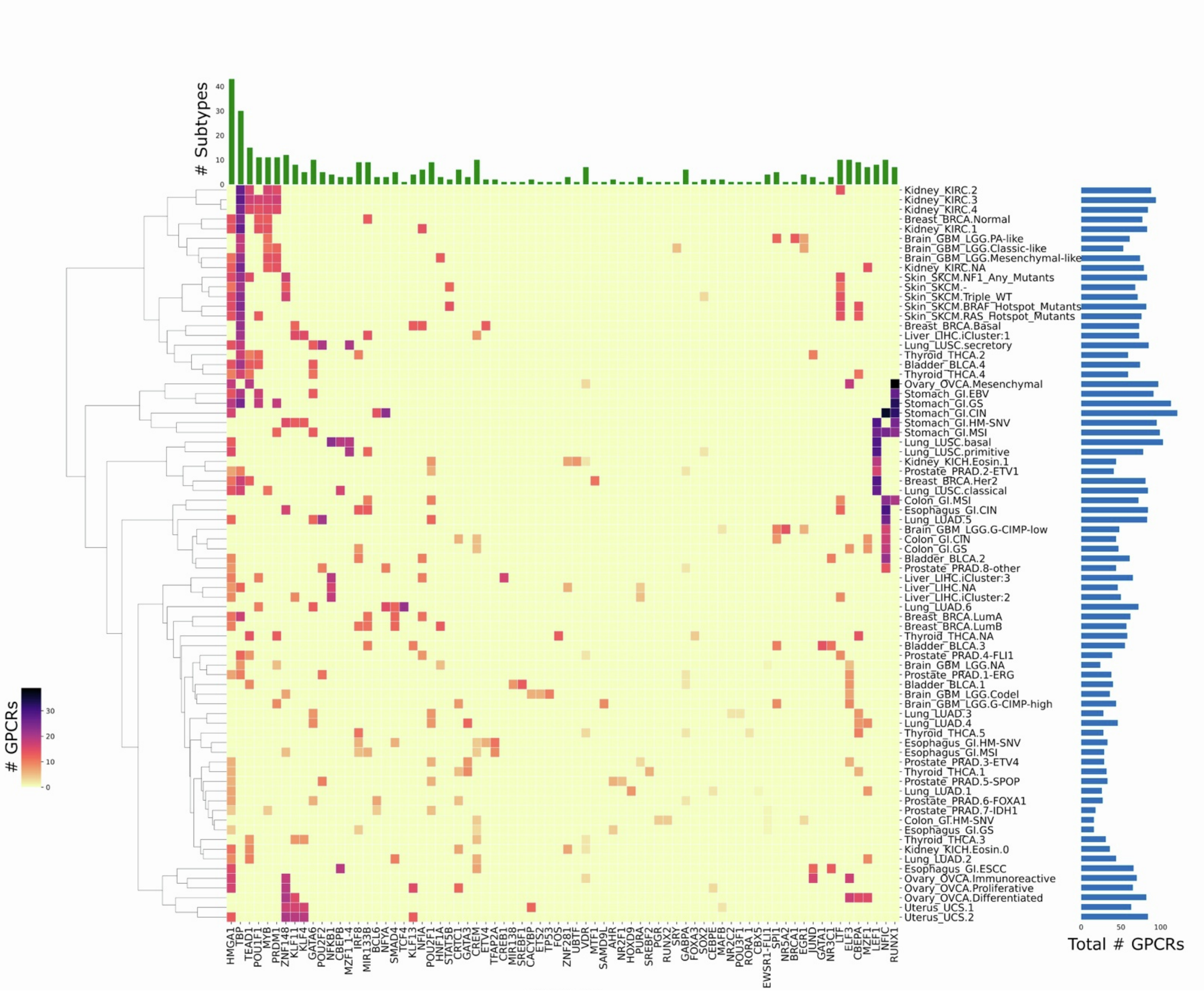

Supplementary Figure S2 Transcription Factor Enrichment, Related to STAR Methods The figure presents a clustermap depicting transcription factor (TF) enrichment based on differentially expressed G protein-coupled receptor (GPCR) genes. The heatmap illustrates co-occurrence patterns, with filled cells indicating TF enrichment in specific cancer subtypes. Color intensity reflects the count of differentially expressed GPCRs associated with each TF in the respective subtype. The barplot atop the clustermap provides the count of subtypes exhibiting TF enrichment. The barplot on the right shows the number of DE GPCRs in the subtype.

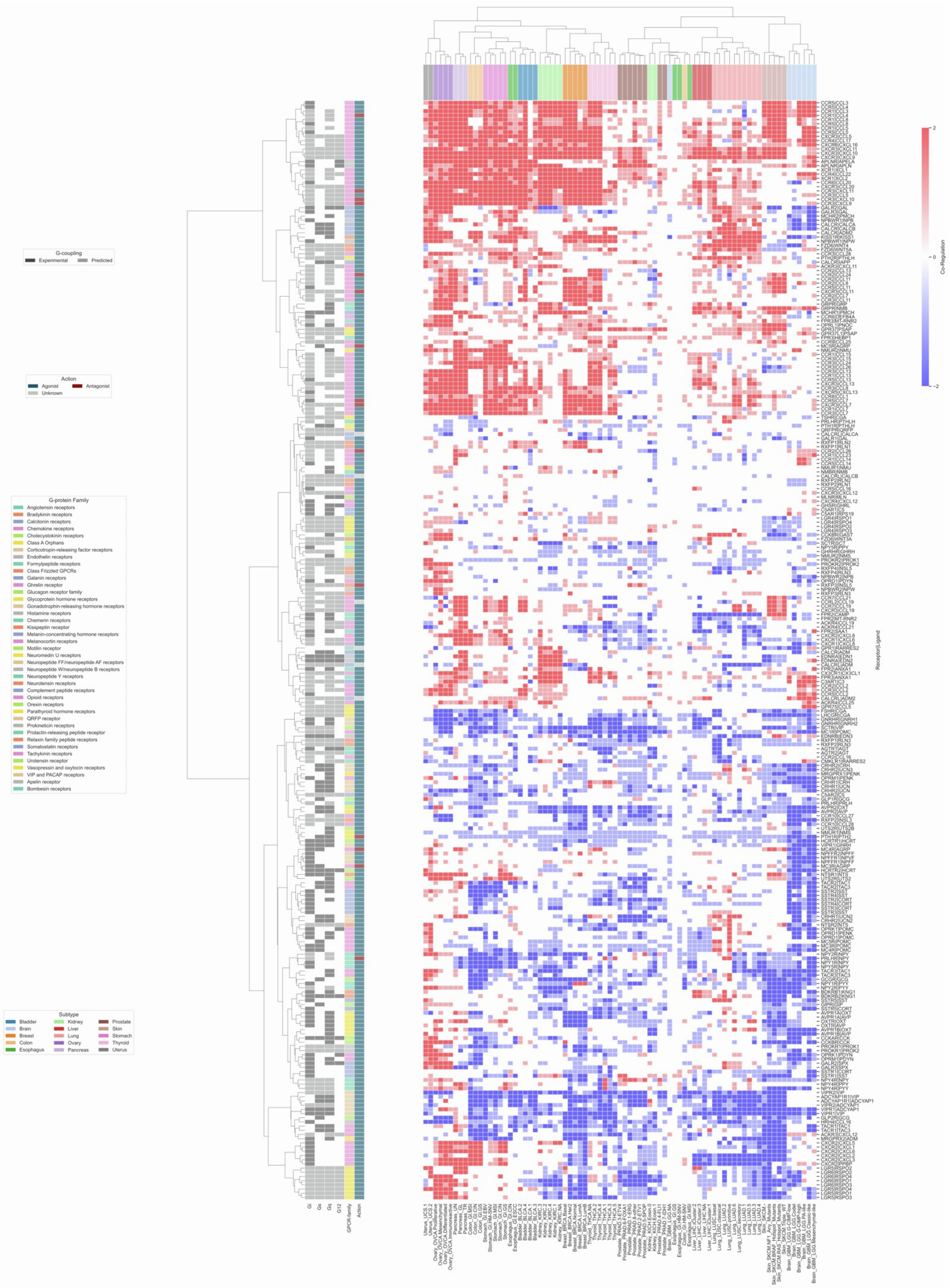

Supplementary Figure S3 Co-differential regulation of GPCR-Ligand pairs in TCGA subtypes, Related to Figure 2 Heatmap displaying the co-differential regulation for Receptor-Ligand pairs across different TCGA subtypes (color-coded at the top row). Darker red represents both receptor and ligand significantly co-up regulated in TCGA (i.e., LFC>1 and BH corrected Padj<0.01) and darker blue represents both receptor and

ligand significantly co-down regulated in TCGA (i.e., LFC<1 and BH corrected Padj<0.01). Paler colors represent either of the receptor-ligand as significantly DE (i.e., |LFC|>1 for both but BH corrected Padj<0.01 for only one of these). White cells indicate anti-regulation or no significant fold change (|LFC|<1) at all in at least one of them.

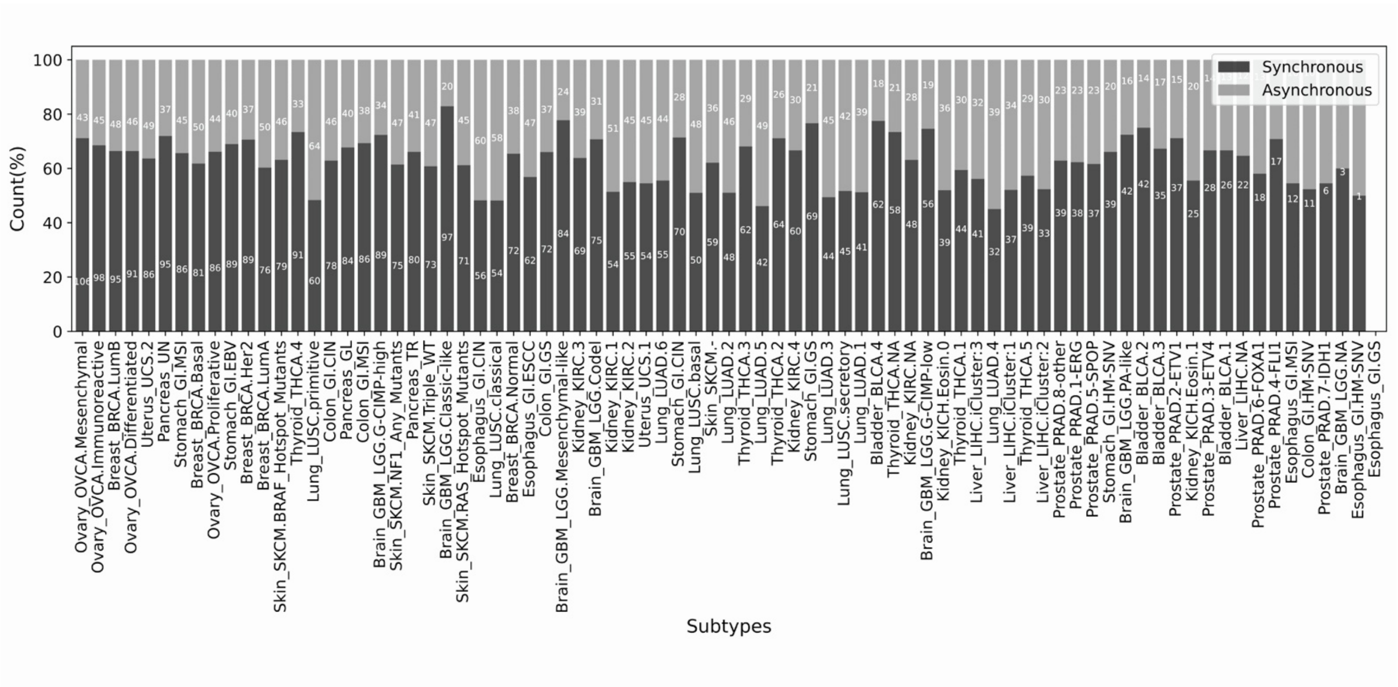

Supplementary Figure S4 Distribution of concordant/discordant co-regulated GPCR-Ligand pairs across subtypes, Related to Figure 2 The barplot represents the proportion of co-regulated GPCR-Ligand pairs (y-axis) across subtypes (x-axis). The concordant (synchronous, with same LFC directionality) pairs are marked in black whereas discordant (asynchronous, with opposite directionality) pairs are marked in gray.

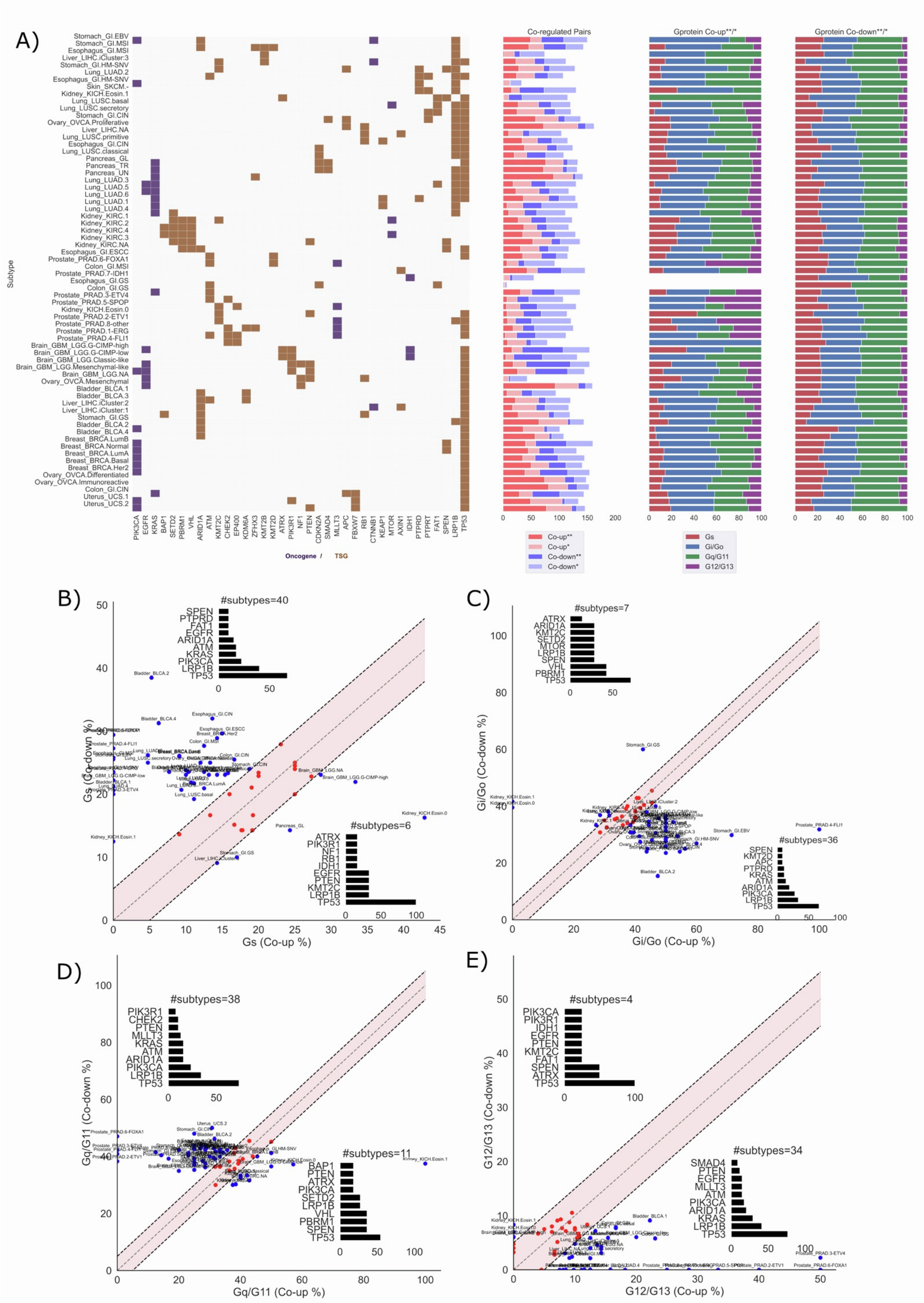

Supplementary Figure S5 Integrated Analysis of cancer gene Mutations, GPCR-Axes, and G Protein Couplings Across Cancer Subtypes, Related to STAR Methods A) The heatmap showcases the specific

patterns of mutated oncogene (purple) and tumor-suppressor (brown) for each cancer subtype. The barplot adjacent to the heatmap represents the subtype specific axes co-regulation information i.e. number of co-regulated receptor ligand pairs, color coded as earlier. Two other barplots on the right provide the proportion of unique receptors (within the co-regulated pairs) which are coupled to either of the four G-proteins: Gs, Gq/G11, Gi/Go and G12/G13. B),C) D) and E) The plots visually depict the imbalance between co-up regulated pairs and co-down regulated pairs across subtypes in lieu of G couplings. Each point represents a cancer subtype. The red shaded area represents a threshold of 5%, which is employed to classify the subtypes in two regions: upper region corresponding to subtypes with higher proportion of G coupled receptors in co-down regulated pairs and vice-versa for the lower region. The inset bar plots show the count of region-specific subtypes with respect to oncogenes, which are ranked accordingly.

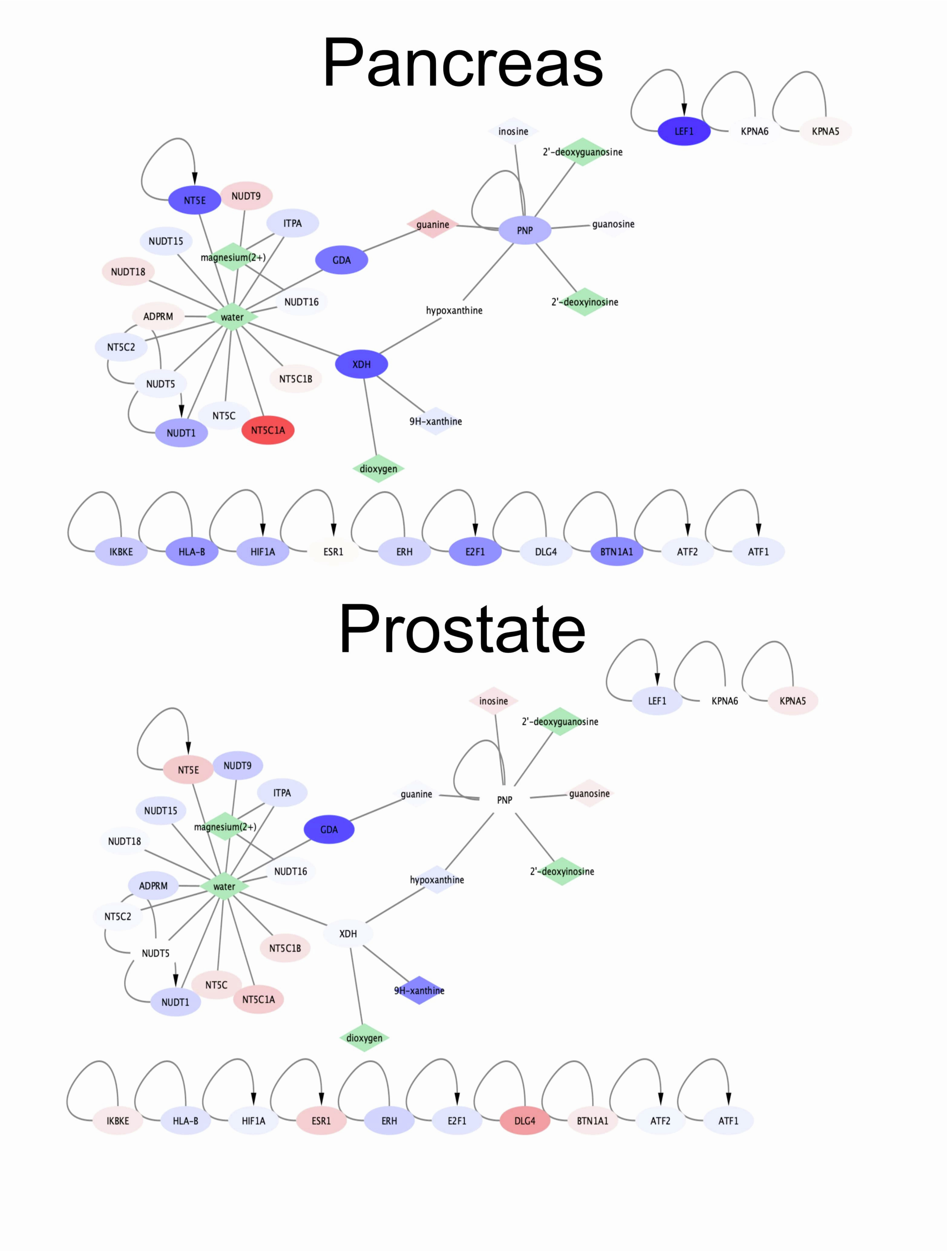

Supplementary Figure S6 Functional interaction network for 'Purine Catabolism' pathway, Related to Figure 3 functional interaction network between genes (ovals) and metabolites (diamonds) in the 'Purine catabolism' pathway in A) pancreatic and B) prostatic cancer. Red nodes indicate upregulated components in cancer, blue nodes indicate downregulated components, and green nodes indicate no information available. The network shows that over-activation of the pathway is contributed by over-expression of both genes and metabolites.

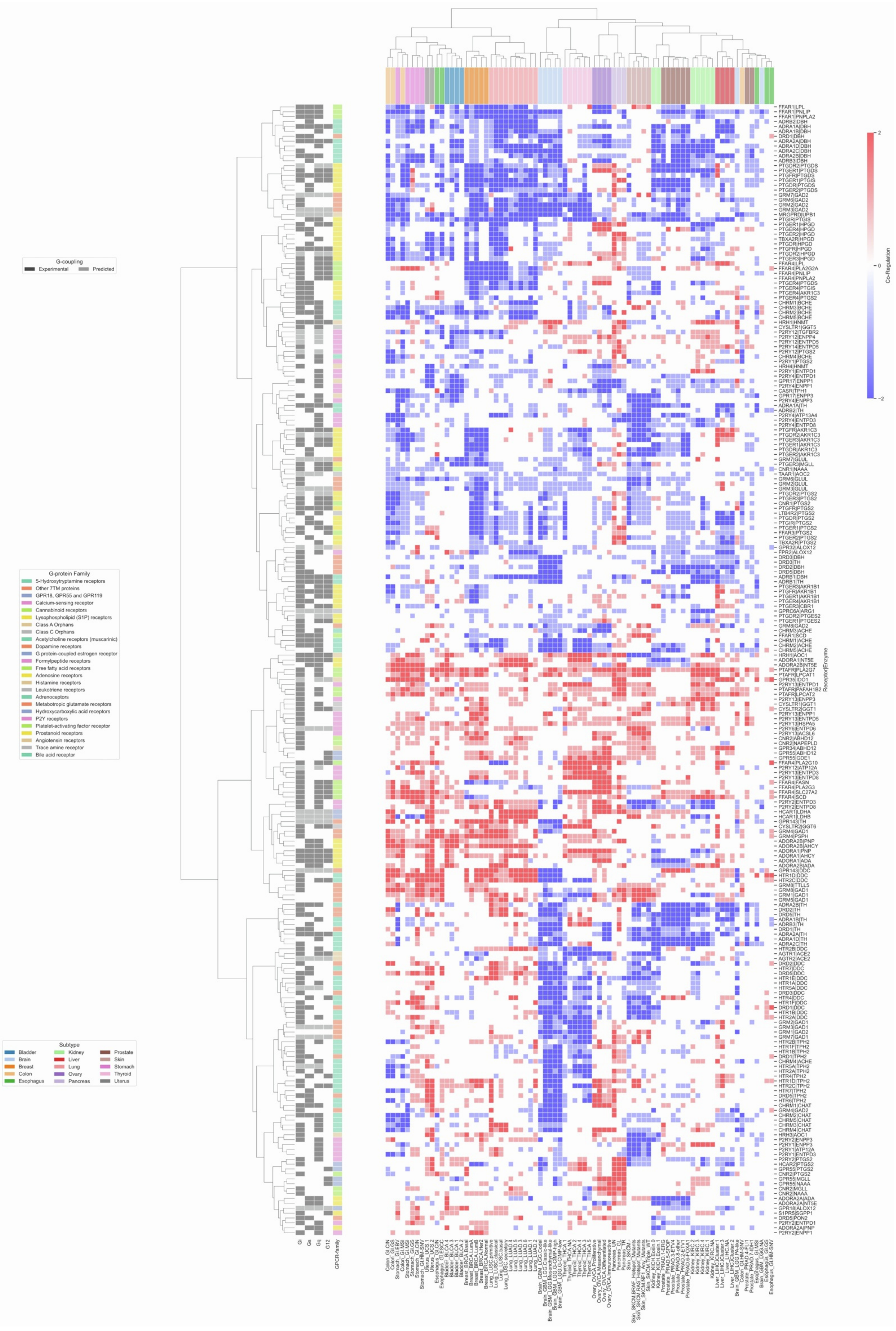

G-coupling
Experimental
Predicted
G-protein Family
5-Hydroxytryptamine receptors
Other 7TM proteins
GPR18, GPR55 and GPR119
Calcium-sensing receptor
Cannabinoid receptors
Lysophospholipid (S1P) receptors
Class A Orphans
Class C Orphans
Acetylcholine receptors (muscarinic)
Dopamine receptors
G protein-coupled estrogen receptor
Formylpeptide receptors
Free fatty acid receptors
Adenosine receptors
Histamine receptors
Leukotriene receptors
Adrenoceptors
Metabotropic glutamate receptors
Hydroxycarboxylic acid receptors
P2Y receptors
Platelet-activating factor receptor
Prostanoid receptors
Angiotensin receptors
Trace amine receptor
Bile acid receptor
Subtype
Bladder
Brain
Breast
Colon
Esophagus
Kidney
Liver
Lung
Ovary
Pancreas
Prostate
Skin
Stomach
Thyroid
Uterus
Gi
Gs
Gq
G12
GPCR-family
Co-Regulation
2
0
−2
Receptor|Enzyme

Supplementary Figure S7 Co-differential regulation of GPCR-Enzyme pairs in TCGA subtypes, Related to Figure 3 Heatmap (center panel) displaying the co-differential regulation for Receptor-Enzyme pairs across different TCGA subtypes (color-coded at the top row). Darker red represents both receptor and ligand significantly co-up regulated in TCGA (i.e., LFC>1 and Padj<0.01) and darker blue represents both receptor and ligand significantly co-down regulated in TCGA (i.e., LFC<1 and Padj<0.01). Paler colors represent either of the receptor-ligand as significantly DE (i.e., |LFC|>1 for both but Padj<0.01 for only one of these). White cells indicate anti-regulation or no fold change at all in at least one of them. Only those pairs which are affected in at least 25% of TCGA subtypes are displayed. A dashed line separating the two clusters, created using Hierarchical clustering, is shown in the middle. Hierarchical clustering was performed using the 'ward' method to identify two homogeneous clusters by minimizing within-cluster variance based on the sum of squared differences of feature values i.e. scores∈[-2,+2] assigned to each pair. Heatmap (left panel) uses color codes to display the ligand's mechanism of action in 'Action', G Protein-coupling associated with the GPCRs and GPCR Family.

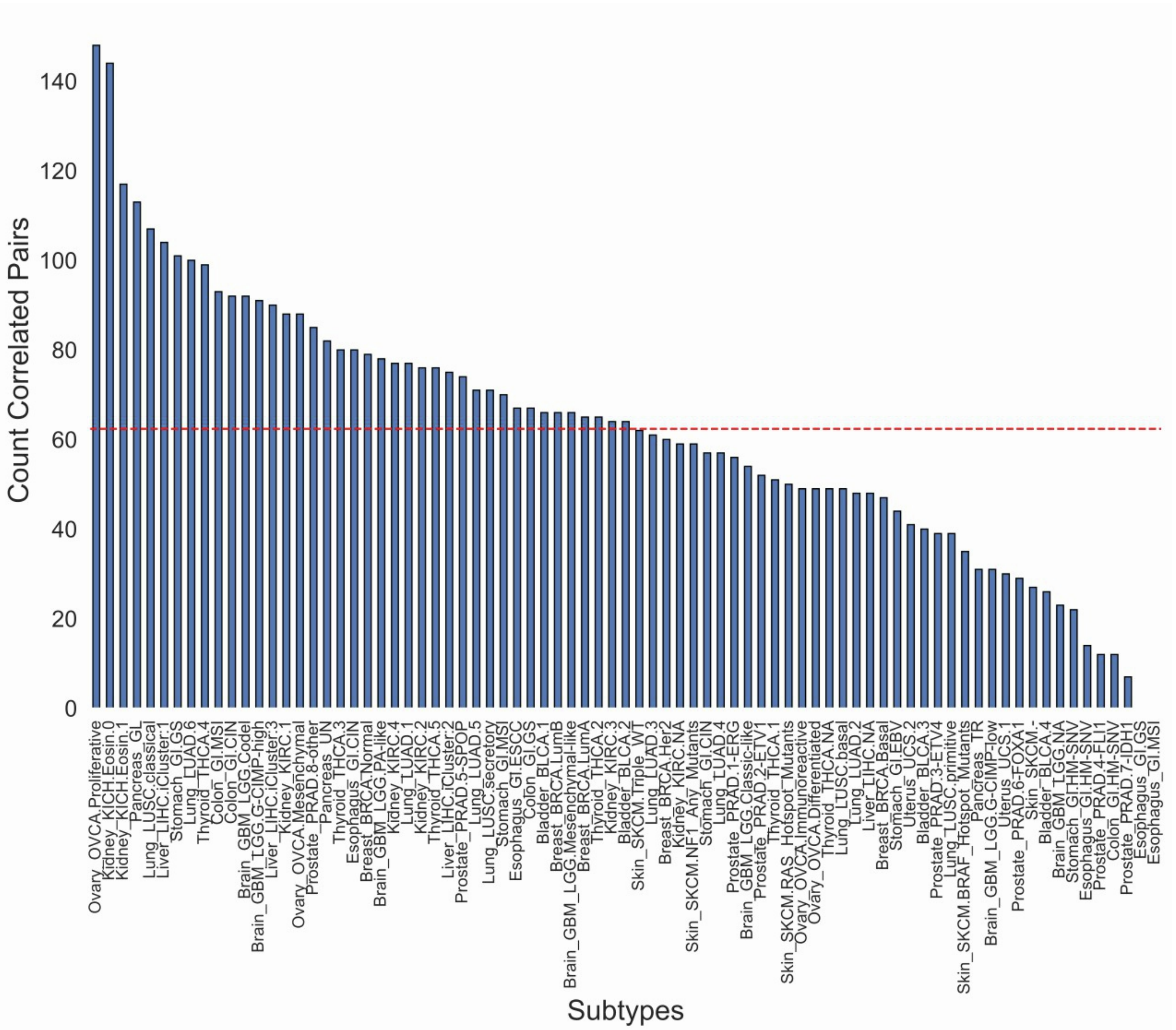

Supplementary Figure S8 Frequency of number of correlated GPCR-Enzyme pairs across subtypes, Related to STAR Methods Barplot with the TCGA molecular subtype-level statistics of the receptor-enzyme pairs with significant receptor-enzyme correlation.

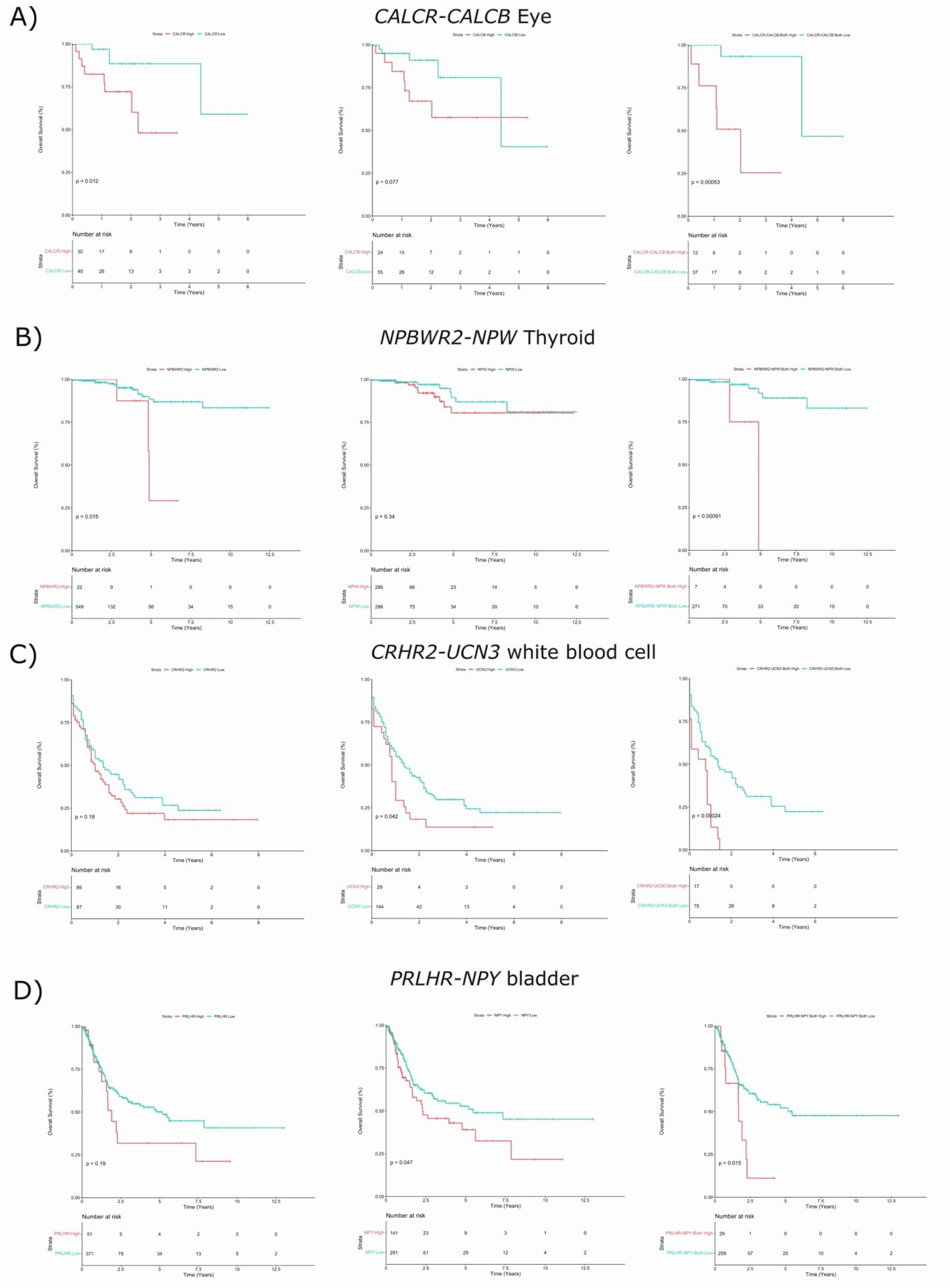

Supplementary Figure S9 Survival Analysis of significant GPCR-Ligand axes in various cancer types, Related to Figure 4 Kaplan-Meier curve for survival analysis done by stratifying patients based on

expression values of representative GPCR-ligand axes, found to be more significantly associated with lower survival than individual components in several cancer types. Each expression group is represented with a colored survival curve, as denoted in the legend. Risk tables below the KM plots represent the number of surviving patients at various time-points. The displayed p-values inside KM plots signifies the outcome of the log-rank test, assessing the statistical distinction in survival between compared groups. The null hypothesis posited was the equivalence of survival curves between groups, indicating identical underlying distributions.

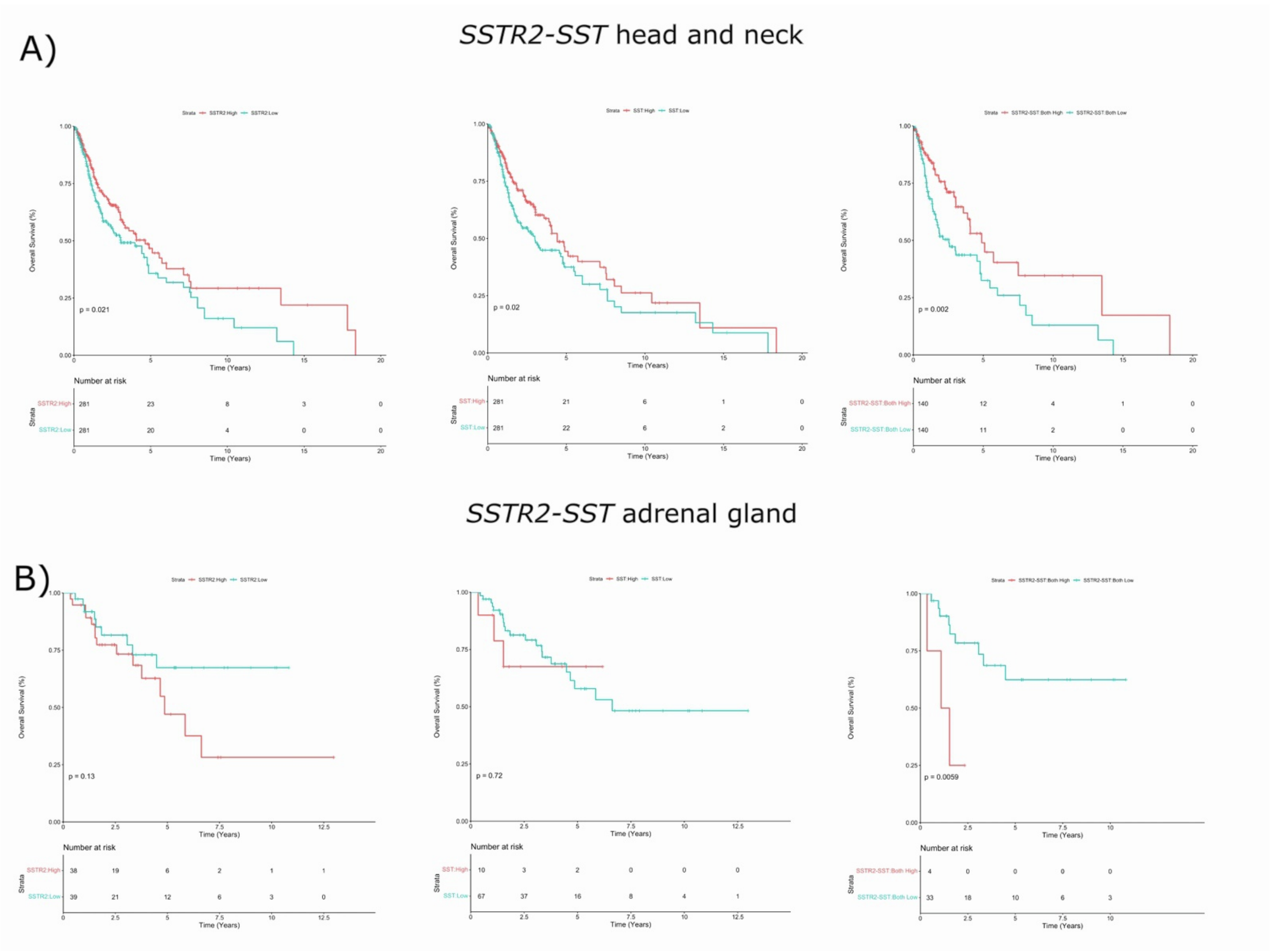

Supplementary Figure S10 Survival Analysis of SSTR2-SST in Head & Neck and Adrenal gland cancers, Related to Figure 4 Kaplan-Meier curve for survival analysis done by stratifying patients based on expression values of SSTR2-SST axis in either head and neck or adrenal gland cancers. Each expression group is represented with a colored survival curve, as denoted in the legend. Risk tables below the KM plots represent the number of surviving patients at various time-points. The displayed p-values inside KM plots signifies the outcome of the log-rank test, assessing the statistical distinction in survival between compared groups. The null hypothesis posited was the equivalence of survival curves between groups, indicating identical underlying distributions.

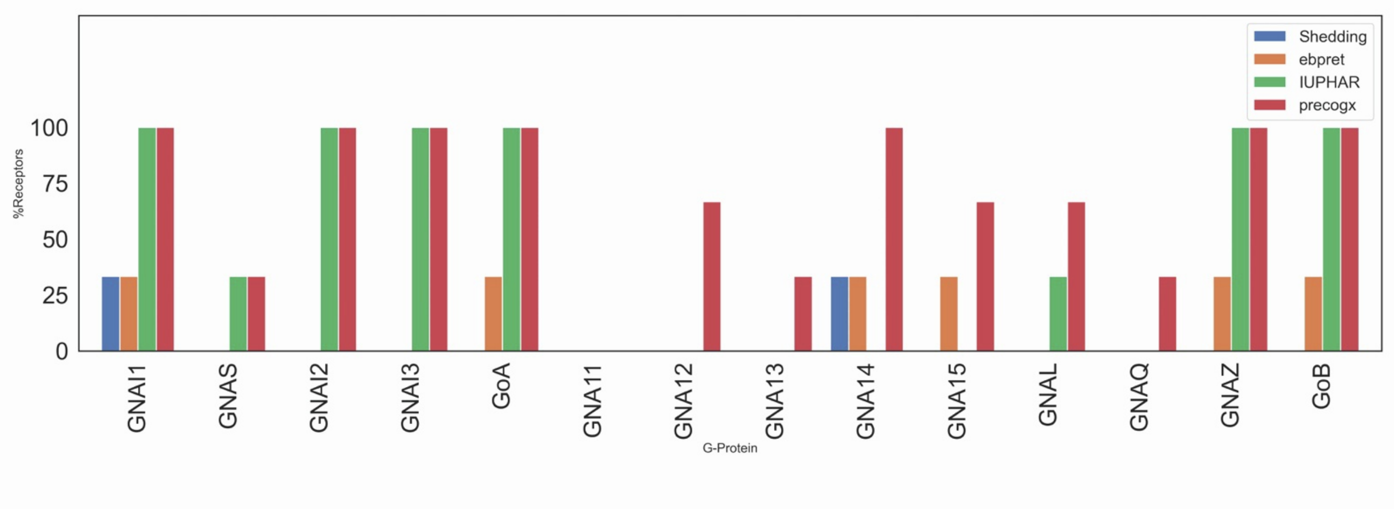

Supplementary Figure S11 G-Protein coupling for survival associated axes in Head and Neck cancer, Related to Figure 4 G protein coupling preferences of axes found significantly associated with higher survival in head and neck: i.e., *SSTR2-SST*, *RXFP3-INSL5* and *RXFP1-RLN3*

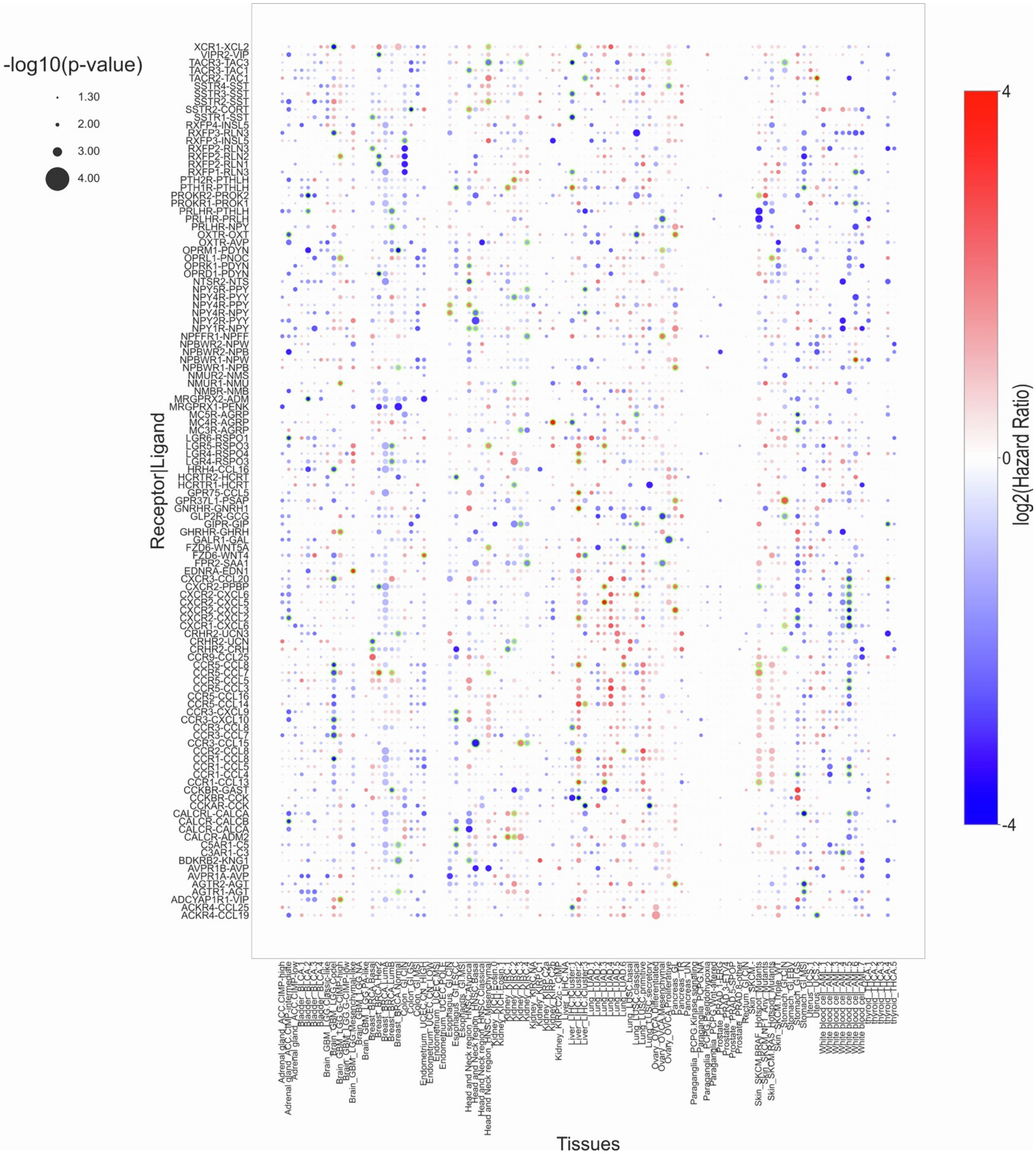

Supplementary Figure S12 Association of GPCR-peptide ligands axes to survival in TCGA molecular subtypes, Related to Figure 4 The bubble plot shows the correlation between the combined-expression levels of GPCR-ligand pairs and patient survival across cancer subtypes. The pairs with a log rank p-value <0.05 and also lower than log rank p-values for individual GPCR/ligand are displayed. Bubble color is proportional to HR: i.e., HR>1 : High expression is correlated with high survival (red); HR<1: High expression is correlated with poor survival (blue). Bubble diameters are proportional to the -log10(log-rank p-value). Green highlighted bubbles represent the most significant instances (sample sizes>5, FDR<0.1).

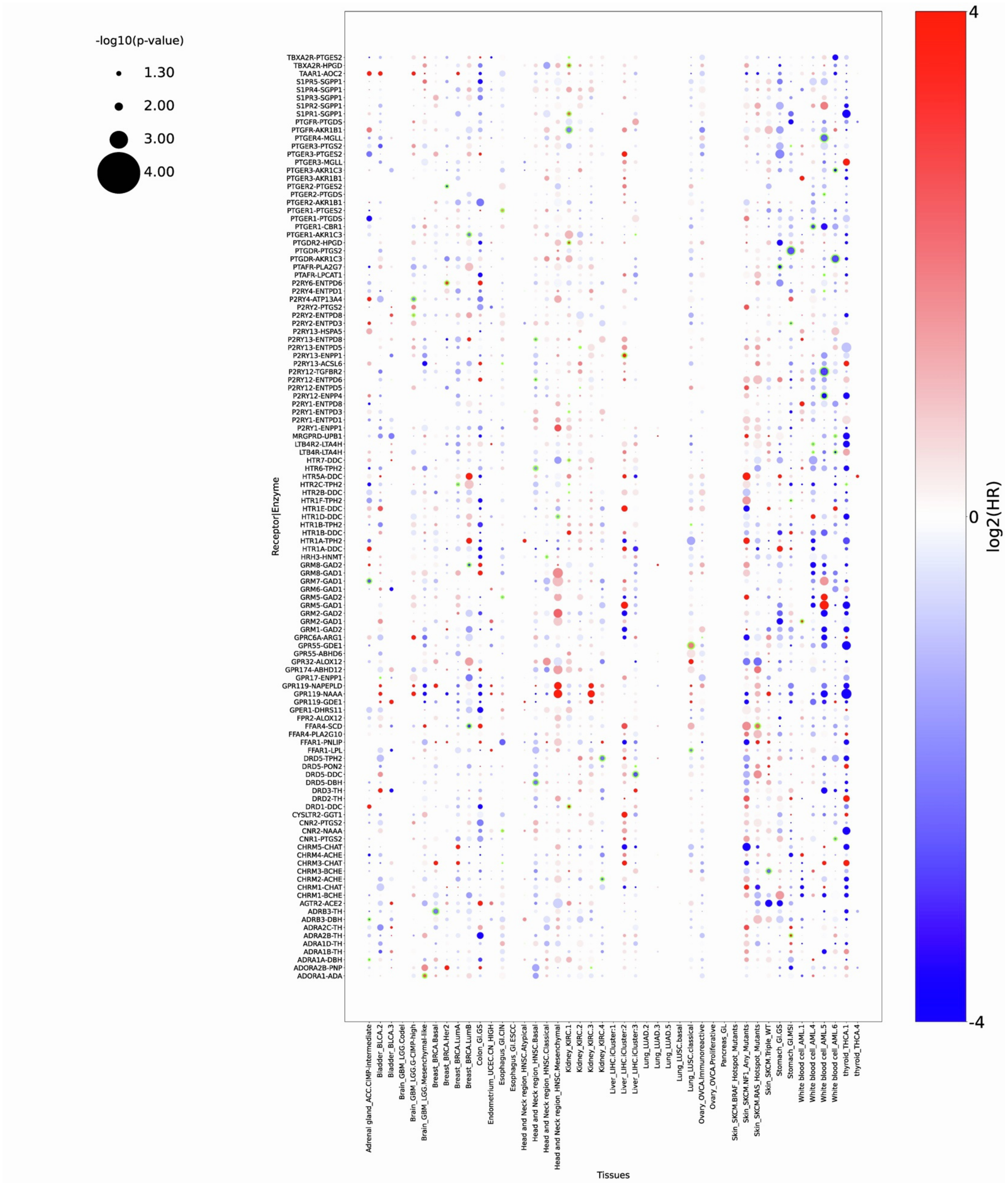

Supplementary Figure S13 Association of GPCR-enzyme axes to survival in TCGA molecular subtypes, Related to Figure 5 The bubble plot shows the correlation between the combined-expression levels of GPCR-enzyme pairs and patient survival across various subtypes. The pairs with a log rank p-value <0.05 and also lower than log rank p-values for individual GPCR/ligand are displayed. Bubble color is proportional to HR: i.e., HR>1 : High expression is correlated with high survival (red); HR<1: High expression is correlated with poor survival (blue). Bubble diameters are proportional to the -log10(log-rank p-value). Green highlighted bubbles represent the most significant instances (sample sizes>5, FDR<0.1).

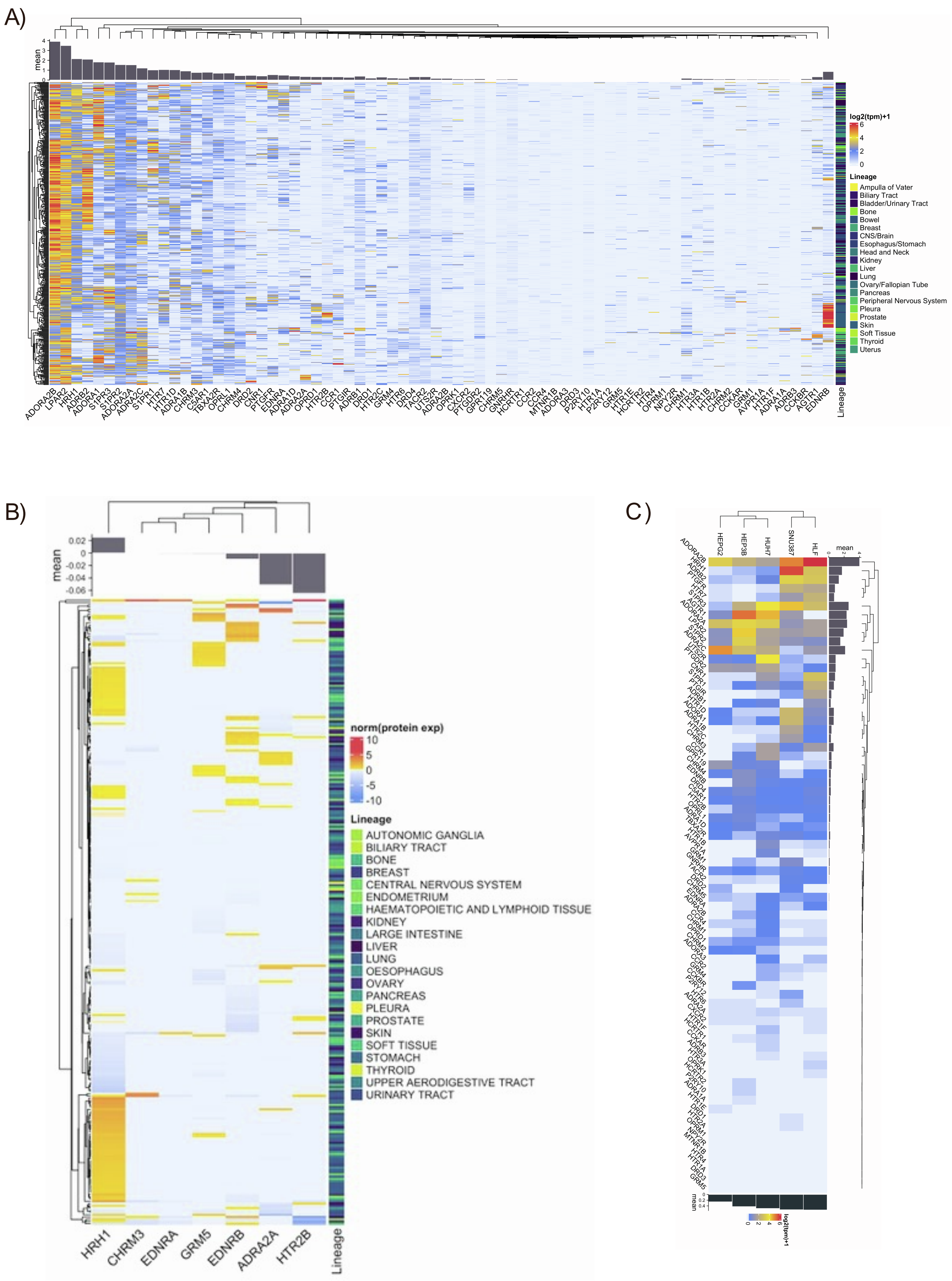

Supplementary Figure S14 Analysis of GPCR expression profiles in PRISM drug screen, Related to Figure 6

A) Analysis of expression profiles in PRISM cell lines of GPCRs targeted by viability-reducing drugs with LFC<-3. Each column represents a specific GPCR, while the rows indicate different cell lines. In particular, ADORA2B emerges as the most highly expressed GPCR. B) The heatmap showcases the relative abundance

of GPCRs targeted by viability-reducing drugs with LFC<-3 at the proteomic level, among them HRH1 is the most highly expressed, in PRISM cell lines. C) In-depth expression profiles of liver cell lines, including HEPG2, used in in vitro experiments. Also, in this subgroup of cell lines, ADORA2B is confirmed to be the most highly expressed receptor.

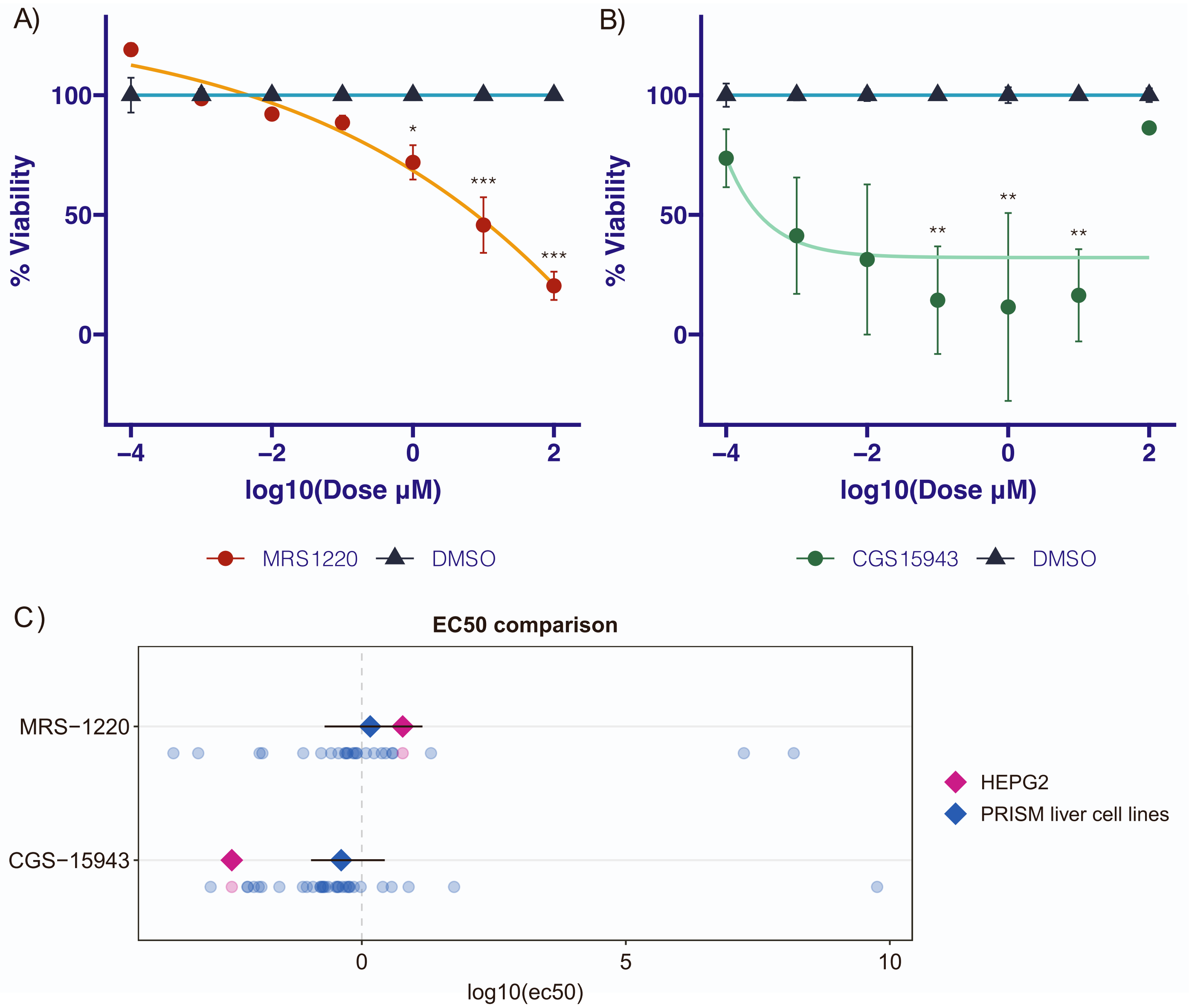

Supplementary Figure S15 HEPG2 cell viability with ADORA2B targeting MRS1220 and CGS15943 drugs, Related to Figure 6 Comparison of viability curves of HEPG2 cells treated with A) MRS1220, B) CGS15943, and the vehicle control (DMSO). The viabilities were normalized to the baseline viability observed with the DMSO. Each curve represents the mean viability, and error bars indicate the standard error of the mean. Statistical significance was assessed using the Kruskal-Wallis rank sum test, highlighting the differences between DMSO and the drugs treated cells in each single concentration (ns $p > 0.05$; * $p \leq 0.05$; ** $p \leq 0.01$; *** $p \leq 0.001$; **** $p \leq 0.0001$). C) EC50 comparison of MRS1220 and CGS15943 between HEPG2 treated cells and liver cancer cells available in PRISM.

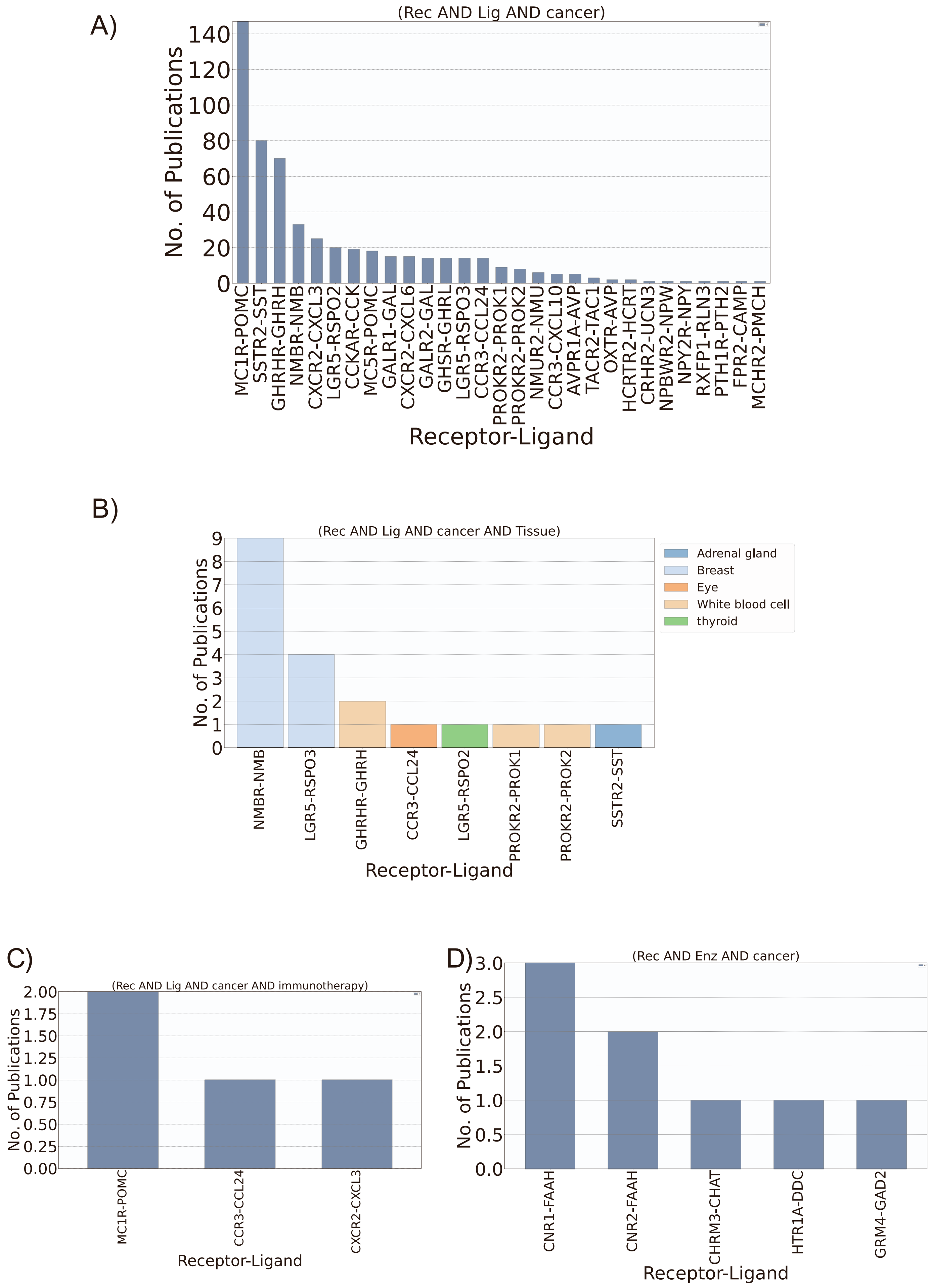

Supplementary Figure S16 PubMed evidence for GPCR-Ligand and GPCR-Enzyme interactions in Cancer, Related to STAR Methods frequency of Pubmed evidences obtained by querying with the following

keywords: A) GPCR name *and* ligand name *and* cancer; B) GPCR name *and* ligand name *and* cancer *and* tissue; C) GPCR name *and* ligand name *and* cancer *and* immunotherapy; D) GPCR name *and* enzyme name *and* cancer
